# Modeling of Wnt-mediated tissue patterning in vertebrate embryogenesis

**DOI:** 10.1371/journal.pcbi.1007417

**Published:** 2020-06-24

**Authors:** Jakob Rosenbauer, Chengting Zhang, Benjamin Mattes, Ines Reinartz, Kyle Wedgwood, Simone Schindler, Claude Sinner, Steffen Scholpp, Alexander Schug

**Affiliations:** 1 John von Neumann Institute for Computing, Jülich Supercomputer Centre, Forschungszentrum Jülich, Jülich, Germany; 2 Living Systems Institute, School of Biosciences, College of Life and Environmental Sciences, University of Exeter, Exeter, United Kingdom; 3 Steinbuch Centre for Computing, Karlsruhe Institute for Technology, Karlsruhe, Germany; 4 Department of Physics, Karlsruhe Institute of Technology, Karlsruhe, Germany; 5 Living Systems Institute, Centre for Biomedical Modelling and Analysis, College of Engineering, Mathematics, and Physical Sciences, University of Exeter, Exeter, United Kingdom; 6 Faculty of Biology, University of Duisburg-Essen, Essen, Germany; Northeastern University, UNITED STATES

## Abstract

During embryogenesis, morphogens form a concentration gradient in responsive tissue, which is then translated into a spatial cellular pattern. The mechanisms by which morphogens spread through a tissue to establish such a morphogenetic field remain elusive. Here, we investigate by mutually complementary simulations and *in vivo* experiments how Wnt morphogen transport by cytonemes differs from typically assumed diffusion-based transport for patterning of highly dynamic tissue such as the neural plate in zebrafish. Stochasticity strongly influences fate acquisition at the single cell level and results in fluctuating boundaries between pattern regions. Stable patterning can be achieved by sorting through concentration dependent cell migration and apoptosis, independent of the morphogen transport mechanism. We show that Wnt transport by cytonemes achieves distinct Wnt thresholds for the brain primordia earlier compared with diffusion-based transport. We conclude that a cytoneme-mediated morphogen transport together with directed cell sorting is a potentially favored mechanism to establish morphogen gradients in rapidly expanding developmental systems.

## Author summary

How entire organisms develop out of single cells is a long-term challenge in the life sciences. Morphogens are crucial signaling molecules organizing cell fates and patterning by their local concentrations. While many morphogens diffuse freely, specialized cell extrusions can facilitate directed cell-to-cell transport for morphogens of the Wnt/β-Catenin family. We performed simulations of quickly growing tissue take this into account, back to back with in-vivo experiments. Our simulations suggest that stochasticity effects lead to non-physiological fluctuating boundaries of tissue regions if not properly controlled. Such control can be achieved via directed cell sorting and apoptosis. We provide experimental evidence for both mechanisms. We observe a distinct temporal difference between the transport mechanisms, with cytonemes facilitating an earlier establishment of a stable pre-pattern. Overall, simulations suggest that cytoneme-mediated Wnt transport is advantageous over diffusion-based transport and a potential general mechanism to establish morphogen gradients in rapidly expanding developmental systems.

## Introduction

Multicellular organisms all face the same fundamental challenge during development: the generation of distinct cell types and organs at specific positions in the body. Using a reaction-diffusion model, seminal work of Alan Turing predicted the existence of form defining chemical species termed morphogens [1]. Morphogens spread with defined transport properties from the site of production and interact with recipient tissue, e.g. by feedback mechanisms such as auto-activation and induction of faster-diffusing inhibitors, leading to the formation of regular patterns such as periodic stripes [2].

The translation of the spatial distribution of morphogens into patterns of multiple cell fates was addressed by Lewis Wolpert in his French flag analogy [3]. The morphogen signal is produced by source cells and spreads through adjacent tissue where the signal is continuously depleted [4]. Thus, the morphogen forms a concentration gradient with a higher concentration closer to the source and a lower concentration in more distant areas. Cells respond to the concentration of this signal via the induction of a morphogen concentration-specific transcriptional code, leading to the acquisition of cellular fates at specific positions in the tissue. In particular, the fate of individual cells is defined by their local morphogen concentration. This fate is defined with respect to thresholds of the concentration that are associated with specific cell types. The obvious challenge for recipient cells is to sense the morphogen concentration and adopt their corresponding fate accordingly–especially if the slope of the gradient is shallow. Accurate morphogen concentration detection is essential for the establishment of a tissue with discrete domains with stable and sharp boundaries.

The Turing model is built on the foundations of symmetry-breaking leading to self-organization, while Wolpert’s French flag model provides a simple, but elegant way to translate a gradient into regional differences essential for pattern formation [5]. Almost all existing gradient models for pattern formation are derivatives and refinements of these pre-eminent models and include the following assumptions: First, linear diffusion is proposed as the general transport mechanism of morphogens. Second, the majority of models focus on slowly changing recipient fields for generation and maintenance of gradients within. A famous, well-established example where these models are applied is Dpp morphogen gradient in the Drosophila wing disc [6, 7]. Recent observations in other signaling systems, however, question the universality of both of these assumptions. Experiments in Drosophila and vertebrates suggest that distribution of morphogens through signaling filopodia—known as cytonemes—are a prerequisite to orchestrate patterning in quickly reorganizing developing tissue [8–10]. Recently, there is also evidence that cytonemes may play a further essential role in cancer progression [11, 12]. Thus, an evolution of existing models is needed to describe the function of cytonemes in patterning in a tissue-specific context.

Once stable morphogen gradients have been established, cell interior gene regulatory mechanisms are stepping in to robustly determine the cell fate. However, the idea of canalization by C. H. Waddington as well the assumptions of the French flag model have been challenged by analysis of the Bicoid morphogen gradient setting the anteroposterior (AP) patterning in Drosophila embryogenesis. An analysis of the state space of the Bicoid gradient and gene expression mechanisms, that together form attractor states, which are stable to account for variability and perturbations in the development. This provides an elegant way of pattern refinement and stabilization in differentially sized embryos. However, this mechanism relies on the existence of additional signaling pathways in downstream interactions in this case by the gap-genes *hunchback*, *Krüppel* and *Knirps*, for each of the three segments [13, 14]. Those feedback interactions require time to be generated and add a further level of complexity to the patterning mechanism by regulatory interactions between target genes which are expressed after thresholding by a French flag model.

The zebrafish neural plate serves as a further well-documented morphogenetic system in which the Wnt/β-catenin pathway acts as a key morphogen specifying the AP axis [15–17]. In zebrafish, the ligands of the Wnt/β-catenin signaling pathway are expressed at the embryonic margin and spread into the neural plate primordium [18]. Neural plate cells close to the margin, exposed to high concentration of Wnt ligands, acquire fate of hindbrain and spinal cord, whereas cells receiving lower concentrations contribute to midbrain and forebrain tissue. Therefore, development of the zebrafish neural plate is a textbook example of the French flag model. However, it is open to question if this model can be applied in a straightforward manner to simulate the Wnt morphogenetic field. Free diffusion of the highly lipid-modified ligands of the Wnt/β-catenin signaling pathway through the aqueous extracellular space is highly unlikely, and Wnt signals have been found to be associated with membranes during signaling [19, 20]. Instead, it has been hypothesized that Wnt is transported on cellular protrusions—termed cytonemes [21–25]. Indeed, live imaging of the intact zebrafish embryo has demonstrated that the main patterning factor Wnt8a is propagated on cytonemes during neural plate patterning in early gastrulation in zebrafish [12, 22].

In addition to the morphogen transport mechanism, one must also consider the effect that the rapid expansion and dynamic organization of embryonic tissue has on the translation of gradients into stable patterns. Based on the expression of brain region-specific transcription factors, we can infer that Wnt-dependent patterning of the zebrafish neural plate occurs within a few hours post-fertilization (hpf) [26]. The expression of the key morphogen Wnt8a starts to be detectable at the embryonic margin at 4 hpf, and a robust pattern with accurate lineage-restriction at the midbrain-hindbrain boundary is established already at 10 hpf [18, 27]. During this short period, the neural plate forms via intercalation of a 3D tissue mass into a 2D single-layered, pseudostratified neural epithelium. Imaging-based analysis suggests high levels of tissue plasticity during the entire zebrafish embryogenesis [28, 29]. We thus conclude that sophisticated spatial migratory processes of individual cells support the ingression and convergent extension of the growing neural plate.

In summary, the Wnt morphogen gradient has to be translated into a robust AP pattern with accurate primordia boundaries [30] in a time period in which the neural plate undergoes massive expansion and cell rearrangements. Recently a detailed mathematical description of cytoneme mediated morphogen deposition and gradient formation has been developed for a one dimensional and static field of cells[31]. However, a model incorporating tissue dynamics, as well as gradient formation exceeding the cytoneme length has yet to be established. The development of a 2D model describing those non-steady-state processes is the aim of the present manuscript.

Here, we develop a simple yet robust model which considers contact-based morphogen trafficking in a highly dynamic tissue environment. We compare simulations for the generation of the Wnt morphogenetic gradient via the contact-mediated case against the case in which Wnt is transported by free diffusion. We find that both transport mechanisms, when averaged over many cells for a 1D representation, lead to deceptively smooth gradients. By investigating the fate of individual cells, however, we find fuzzy boundaries between the three brain primordia. This includes a significant number of cells adopting different fates to their neighbors in the same region–termed outliers. This observation is in stark contrast to observed sharp regional boundaries in the neural plate. Which mechanisms could sharpen the boundaries? We provide experimental evidence for a cell-sorting mechanism based on the Wnt/β-catenin activity level of single cells during the early patterning phase and increasing apoptosis in a later phase to eliminate occurrences of outliers with fates that are incongruent with the one of the neighboring cells. Implementing both cell sorting and apoptosis in the simulations leads to the formation of refined boundaries. Remarkably, these actions are similarly important for diffusion-based and cytoneme-based transport mechanisms. Our simulations and *in vivo* validation, however, suggest that a cytonemal transport model requires significantly less time to generate a steeper gradient and thus sets more distinct thresholds in the tissue compared to the diffusion-based model. An early and stable gradient is an essential prerequisite to trigger appropriate cell fate acquisition which allows to establish a stable pattern in the rapidly changing zebrafish neural plate.

In summary, we develop and test a stochastic mathematical model to simulate the generation of a morphogenetic field based on cell contact-mediated ligand transport, which can be translated to a reliable tissue patterning at a single cell level in quickly expanding tissue. The application of this model suggests a cytoneme-based pre-patterning phase which requires high tissue plasticity, followed by a refinement phase with directed cell sorting and apoptosis as fundamental principles in patterning of quickly growing tissues (cf. graphical abstract S1 Fig).

## Methods

We choose Wnt/β-catenin-dependent neural plate patterning in the zebrafish to investigate the differences of a cytonemal morphogen transport and a diffusion-mediated transport of morphogens with regard to pattern formation in a rapidly changing tissue. Based on measurements of the zebrafish neural plate, we represent the tissue by a two-dimensional growing domain with an initial length of 60 μm and a width of 1000 μm. Three conceptually distinct processes are making up the main constituents of the model used here (for a graphical model description cf. Fig 8). **Morphogen dynamics** models the deposition, migration and depletion of Wnt within the tissue. The deposition can be modeled by two different mechanisms, diffusion and discrete deposition. **Tissue dynamics** is the process, that describes the dynamic development of the individual cells within the tissue as well as the global tissue expansion by cell division and intercalation of cell layers. **Cell fate determination** classifies the individual cells into cell fates, namely hind-, mid- and forebrain fate based on the obtained Wnt concentrations.

There are multiple models that are used for the simulation of tissue [32–34]. Here, we perform the simulation on a precomputed discrete two-dimensional grid of densely packed circles on random non-overlapping positions, which represent fixed possible positions of cells. We build on the simulation framework from [22]. In zebrafish, morphogenesis occurs during gastrulation on top of the spherical yolk. However, the effects of the curvature of the tissue are disregarded due to the near radial symmetry of the problem. Computations are performed on a non-periodic grid of 1000x1000 μm, in which each cell occupies a circle with an 8 μm radius. Cell movement as well as morphogen transport is confined to the grid size.

In our simulations, we assume: (i) Producing cells are the only source of Wnt, (ii) producing cells produce Wnt continuously, (iii) receiving cells can only sense their own local concentration of Wnt, without knowledge of their global position or orientation, and (iv) cells decide solely based on Wnt concentration thresholds on their fate.

We perform a discrete-event simulation, which is a Monte-Carlo style simulation [35]. Picking a timestep for our Monte-Carlo simulation provides a natural timescale for our model. Two different cell types are distinguished. Two layers of morphogen producing cells at one border of the grid are the morphogen source, the average length of filopodia is 17μm, so filopodia from a third layer would rarely reach receiving cells. Instead of being pushed by the growing tissue, the producing cells are modeled to be static, do not participate in the tissue expansion and do not receive morphogen. We look at the tissue “from their perspective” and move all other cells relatively, i.e. we reset the frame of reference analogous to a Galileo transformation. The morphogen receiving tissue expands due to cell divisions and insertions from the intercalation of the (not explicitly modeled) other cell sheets above and below. The receiving cells can only accept and store morphogen but cannot transfer it to other cells. Five processes are consecutively performed on each cell in every time step. These five possible events are:

- Production
- Tissue growth
- Migration
- Decay
- Apoptosis

The simulation runs through all cells in each time step (Δt = 1s) and checks for possible events. For each event, experimentally measured rates p_event_ are used. In each time step of the simulation, an event is accepted or discarded by comparing the rate of the event to a random number uniformly sampled Rnd∈ [0…1] (acceptance for Rnd<p_event_).

Pseudocode for the simulations and simulation parameters are provided in the SI. The model is implemented in Python v2.7.12 using the libraries numpy v1.14.2 and scipy v1.0.1.

### Modeling of the tissue expansion

We model a two-dimensional cell sheet which is a projection of a three-dimensional tissue on top of the spherical yolk. The tissue expansion and cell dynamics are implemented in the simulation by two mechanisms describing three processes. Receiving cells can be inserted by (1) intercalation or (2) cell division at a specific site. Cell migration (3) is implemented as nearest neighbor exchange. Cell insertions occur due to the thinning of the tissue during epiboly with probability p_ins_. For a cell insertion, a path to the nearest empty grid space is constructed and cells with their morphogen content are successively moved along that path (for visualization see SI cell insertion). The emerging empty space is then filled with a new cell. We assume a similar morphogen distribution in cell sheets above and below during intercalation. So, this new cell inherits its morphogen content and fate from a randomly chosen cell of the same distance ±6μm from the producing cells.

Cell division is implemented as a random event. This leads to similar statistics as the mitosis cycle with cell division after a certain lifetime. Cell division is modeled by the same mechanism (and a joint probability) as intercalation, since in a two-dimensional cell sheet both processes result in the same basic movement in the simulation. The morphogen content of the new emerging cell is still copied from one randomly chosen cell with similar distance to the producing cells.

The rate p_ins_ jointly treats both mechanisms, division and intercalation, with intercalation being the dominant contribution. The parameter is chosen so that the expansion of the tissue in our simulation reproduces the tissue expansion observed in the experiments. A constant probability yields an exponential tissue growth, experimentally we see a growth from 90 μm to 600 μm between 4–8 hpf, this is reproduced by the choice of our parameters producing an assumed exponential cell growth.

Notably the final size of the tissue in our simulations can vary depending on the initial insertions. Since insertion events are purely random, fluctuations at early times of the simulation have large impact on the final size of the tissue.

Cell migration is implemented as a switching of sites with a uniformly randomly chosen nearest neighbor (nearest five cells) with probability p_mig_.

### Modeling of the morphogen transport

Assuming evolution could select for different transport mechanisms, we compare the effects of a cell contact-mediated propagation mechanism by cytonemes with a contact-independent, diffusion-based trafficking and implement them as exclusive mechanisms in separate sets of simulations. Cells cumulatively collect Wnt content and independent of the transport mechanism passively carry morphogen with them due to migration. This is active for both transport mechanisms and the only way to enable a gradient longer than the filopodia length for the cytoneme based transport.

We implement the cytoneme-based transport as a stochastic process in which cytonemes establish a direct contact between Wnt producing and Wnt receiving cells with an experimentally measured rate p_fil_. Based on our quantifications [22], cytonemes are able to deposit Wnt over an average distance of 17 μm, which represents about two cell layers. The length and angle (relative to vertical) of the formed filopodium are randomly sampled from the respective measured empirical distribution [22] (also see SI). A filopodium then deposits morphogen in a receiving cell if its tip ends up in proximity to a cell’s surface (2 μm), otherwise it is retracted. Each filopodium attempts one deposition and is afterwards deleted. The time for a filopodium to form and grow to its final length is incorporated into the formation rate.

The diffusion-based transport is implemented using Reynold’s transport theorem for growing domains to account for the fast expanding tissue [36] in one dimension. The theorem respects the dilution of the extracellular morphogen concentration C by tissue expanding with the growth field $u=\frac{dx}{dt}$ [36]. The differential equation is

$$\frac{\partial }{\partial t}C+u\frac{\partial }{\partial x}C+C\frac{\partial }{\partial x}u-D\frac{\partial^{2}}{{\partial x}^{2}}C=0$$

numerically solved in each time step.

The growth field u describes the advection of the morphogen due to the expanding tissue and can be determined using the tissue length *L*(*t*) = *L*_0_∙*e^κt^* by

$$u=\frac{dx}{dt}=\frac{\frac{\partial L(t)}{\partial t}}{L(t)}=\kappa$$

Therefore, u is a constant that is defined by the expansion of the tissue and determined by the experimentally seen expansion of the tissue. We assume *L*(0) = 60 and *L*(9000) = 1000 which yields *u* = 0.0003.

The diffusion constant D is set to 0.1 μm2/s [36, 37], unless stated otherwise. The morphogen content of the individual receiving cell is increased by the local morphogen concentration C (x, t) in each time step. The morphogen absorption by the cells is assumed to be small and without influence on the extracellular morphogen concentration and is thus not included in our model. Boundary conditions at the origin are set to *C*(*x* = 0,t) = 0.1. (For a parameter scan of C(0) and D see S6 Fig).

In both mechanisms the morphogen content decays with a probability of p_decay_ within the cells by decreasing the cumulatively acquired morphogen content by 1.

### Patterning

As a second part the patterning of the tissue is evaluated. This is done by analyzing the Wnt contents which were cumulatively obtained in cells during the first step. Cells acquire their individual fate based on the Wnt concentration they receive relative to specified threshold values. The thresholds are fixed values that translate the absolute Wnt concentration of a cell into its cell fate. It is difficult to find out that exact value since it is unclear at what time the final fate is acquired. The thresholds are therefore defined a specific time t_TRS_ during the simulation and are chosen so that the number of cells acquiring each of the three cell fates is the same. The thresholds in our model are fixed values that are intrinsic properties of the cells and the time t_TRS_ used is given in the figure captions. The effects of the time t_TRS_ at which the thresholds are set is addressed in Fig 5.

### Directed migration

We introduce a probability gain for a cell with high Wnt concentration to switch with a cell with low Wnt concentration towards posterior direction, namely towards cells with high Wnt activity level to the random neighbor swapping introduced earlier. In addition, we introduce a probability gain for a cell with low Wnt concentration to switch with a cell with high Wnt concentration towards the anterior—in the direction towards cells with low Wnt activity level. In each migration attempt with the probability p_mig_ the morphogen content of the considered cell and the neighbors is compared. Directed migration is introduced by a factor p_dirmig_ which favors the exchange of cells that reinforce the concentration gradient, dependent upon the difference in morphogen content (for more information see SI directed migration). With increased probability the considered cell preferentially switches places with a cell closer to the producing layers if it has higher morphogen content and moves away from the producing layers if it has a lower morphogen content.

### Apoptosis

If the Wnt content of a single cell strongly differs from the averaged content of all five nearest neighbors, an apoptotic event can occur. The threshold is tuned so that between 100 and 130 cells, corresponding to about 2.8–3.7% of all cells, undergo apoptosis, this corresponds to the experimentally found values (cf. Fig 4). Each of these cells is discarded and replaced by a random cell with a similar (±6 μm) distance to the producing cells.

Both processes, directed migration and apoptosis can be individually enabled or disabled for each simulation.

### Experimental method

The injection of mRNAs is performed as described in the following: 150 ng of capped and in vitro transcribed mRNA (mMessage Machine Kit, Ambion) for Gsk3β-GFP and Gsk3β-DN-GFP is microinjected into one cell at the 8 blastomere stage to generate cell clones [38]. Embryos are incubated at 28°C and subjected to microscopical analysis at sphere stage (4 hpf). For the following confocal analysis, live embryos are embedded in 0.7% low melting Agarose (Sigma-Aldrich) dissolved in 1 Ringer’s solution. Images are obtained with a Leica TCS SP8 X confocal laser-scanning microscope using 63x dip-in objective. Image processing is performed with Imaris 8 software (Bitplane AG, Switzerland).

To block Wnt secretion, zebrafish embryos are treated with IWP12 (5 μM; 1%DMSO v/v; SigmaAldrich) from the indicated time points to 10 hpf [39]. Embryos are incubated at 28°C and fixed for whole-mount mRNA in situ hybridization (ISH) or antibody staining at indicated stages. For in-situ hybridization, digoxygenin-labelled probes are prepared from linearized templates using an RNA labelling and detection kit (Roche) [40]. For detection of apoptotic cells, whole-mount immunofluorescence of Caspase3 (1:500 anti rabbit anti active Caspase3, 0.5mg/ml, Clone 92–605, BD Pharmigen) is performed [41]. Embryos are transferred to 70% Glycerol/PBS (v/v). Images are taken on an Olympus SZX16 microscope equipped with a DP71 digital camera by using Cell A imaging software.

All animal work is approved by the by the GM safety committee of the University of Exeter and the Home Office by appropriate licences as specified in the following: S Scholpp's project licence (PPL) No P5FA1DA44, Use of Animals (Scientific Procedures) Act ASPA 1986, Jun 2017-Jun 2022 and the personal licences (PILs) awarded to S Scholpp (IC325CEA2). The number of embryos used are defined in accordance to the "three R" rule and the optimal number of embryos for each experiment has been carefully evaluated. An average of 30 embryos/larvae are used per condition inside each experiment. Statistical analysis is performed to minimise the number of embryos/larvae required to deliver significant results. This analysis varies with the experiments undertaken.

## Results and discussion

### Initial simulations

**Fig 1:**
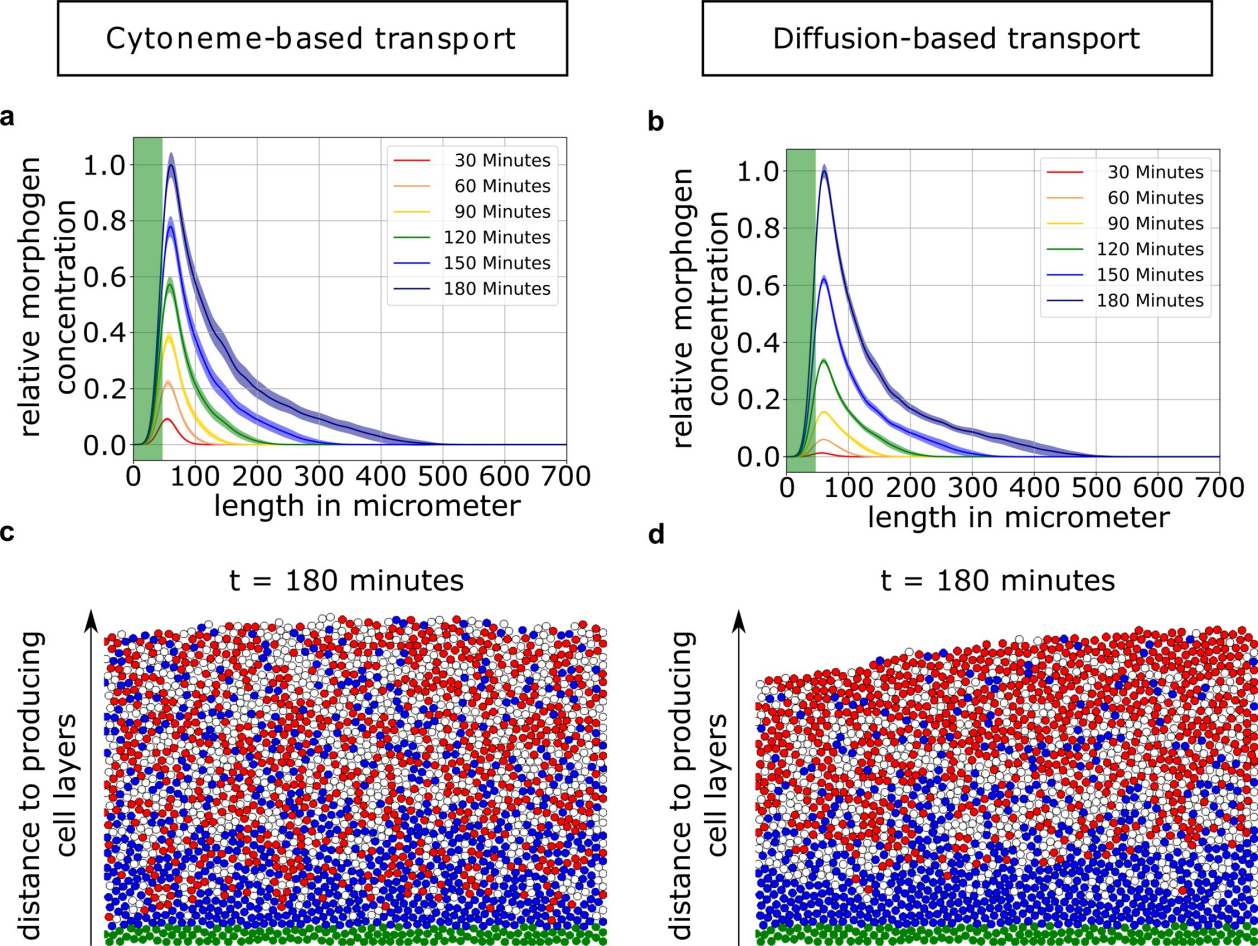
Comparison of cytoneme-based and diffusion-based Wnt transport. **a)** and **b)** show the mean gradient of Wnt concentration (100 simulations) with standard deviation in transparent over the expanding tissue at different times (normalized to the maximum at *t* = 180 min) with respect to the distance from the embryonic margin, the green area indicates Wnt producing cells. **c)** and **d)** show the corresponding 2D cell distribution at the final stage (*t* = t_TRS_ = 180 min). Cell fates are determined by thresholds on the local single cell Wnt-concentration. The thresholds are set at t = 180 min so that the cell-numbers in forebrain-, midbrain- and hindbrain-fate are equal. Besides the Wnt producing cells shown in green, the colors of the cells represent different cellular fates: forebrain fate is indicated in red, midbrain fate in white and hindbrain fate in blue.

First, we investigate the averaged Wnt concentrations within the tissue (Fig 1A and 1B) to obtain the 1D gradients. Despite the short signaling range of the cytonemes, concentration gradients spreading over the whole tissue form due to the expansion of the receptive field (Figs 1A and 1B; 5). The graphs of the concentration gradients produced by either transport mechanism seem suitable to account for patterning and support the French flag model [22]. Such a 1D perspective on the tissue simulations, however, averages out single-cell Wnt concentrations. This obfuscates the underlying complex tissue structure of individual cells originating from stochasticity effects. The individual cell is critical since the fate decision is made on a single cell level and based on the local morphogen concentration. In contrast to the 1D averaged gradients, simulations display great variability in the distribution of cell fates in the two-dimensional cell sheet—regardless of the transport mechanism (Fig 1C and 1D).

For the diffusion-based mechanism (Fig 1D), variability in the distribution of cell fates in the 2D representation is surprising as one might expect smooth transitions of cell fates, since diffusion of Wnt molecules in the extracellular space leads to a smooth gradient field. This exposes recipient cells at the same distance of the source to the same Wnt (extracellular) concentration for uptake. The main cause for the occurrence of mixed cell identities arises from the highly dynamic cell migration processes during the expansion period with the cells intermingling after the acquisition of morphogen. In these simulations, the additional stochastic deposition of Wnt via cytonemes to the receiving cells results in even more strongly intermingling cell fates (Fig 1C and 1D).

### Experimental evidence for cell sorting mechanisms

So far, the results of our simulations do not reproduce the experimentally observed separated regions of cell fates with sharp boundaries. To address this, we consider cell sorting processes that could mitigate variability in the spatial distribution of cell fates. For example, adhesion changes can drive sorting of individual cells as observed in the abdomen of Drosophila [42], at rhombomere boundaries in the hindbrain [43, 44] and in the spinal cord in zebrafish [45] just to name a few. The main cell responses suggested to trigger cell sorting are contact-dependent cell repulsion, differential cell adhesion and cortical tension due to physical barriers [46]. Cells of adjacent segments are able to self-organize if the adhesion strength between the segments differs. Based on this idea, we explore the influence of directed cell sorting on patterning in the primordium of the neural plate.

First, we investigate single cell dynamics during early zebrafish development via a time-resolved lightsheet microscopy dataset [47]. The dataset provides the positions of cells’ centers and their corresponding three-dimensional trajectories during zebrafish gastrulation. We observe that, during this process, cells intercalate to form a pseudo-epithelial cell sheet. To quantify the cellular dynamics, in particular cell relocation, during this process, we measure the duration in which individual cells retain the same neighboring cells and find that the majority of cells maintain contact with the neighboring cells for less than 10min (cf. S3 Fig). We propose that cells exchanging their position with neighboring cells, as a result of the mesenchymal character of the neural plate progenitors [48].

Second, we consider whether the observed highly dynamic changes of cell positions within the tissue is influenced or even controlled by the Wnt morphogenetic gradient. Indeed, preferential mixing of the cells is a mechanism that can establish patterns and stabilize boundaries in a morphogenetic field. In particular, Steinberg’s differential adhesion hypothesis predicts that cell sorting can generate distinct cell aggregates [49].

β-catenin is the central component in the Wnt signaling cascade and plays a pivotal role in cell adhesion [50]. It was initially characterized in Drosophila as the segment polarity gene, Armadillo, with an essential function in cell adhesion. In the adhesion complex, β-catenin and α-catenin are associated with cadherins at the plasma membrane and provide a stable link to the cytoskeleton [51]. Accordingly, we ask if directed cell sorting based on the β-catenin level can act to correct the imprecision within the morphogenetic system in a dynamic tissue such as the neural plate.

**Fig 2:**
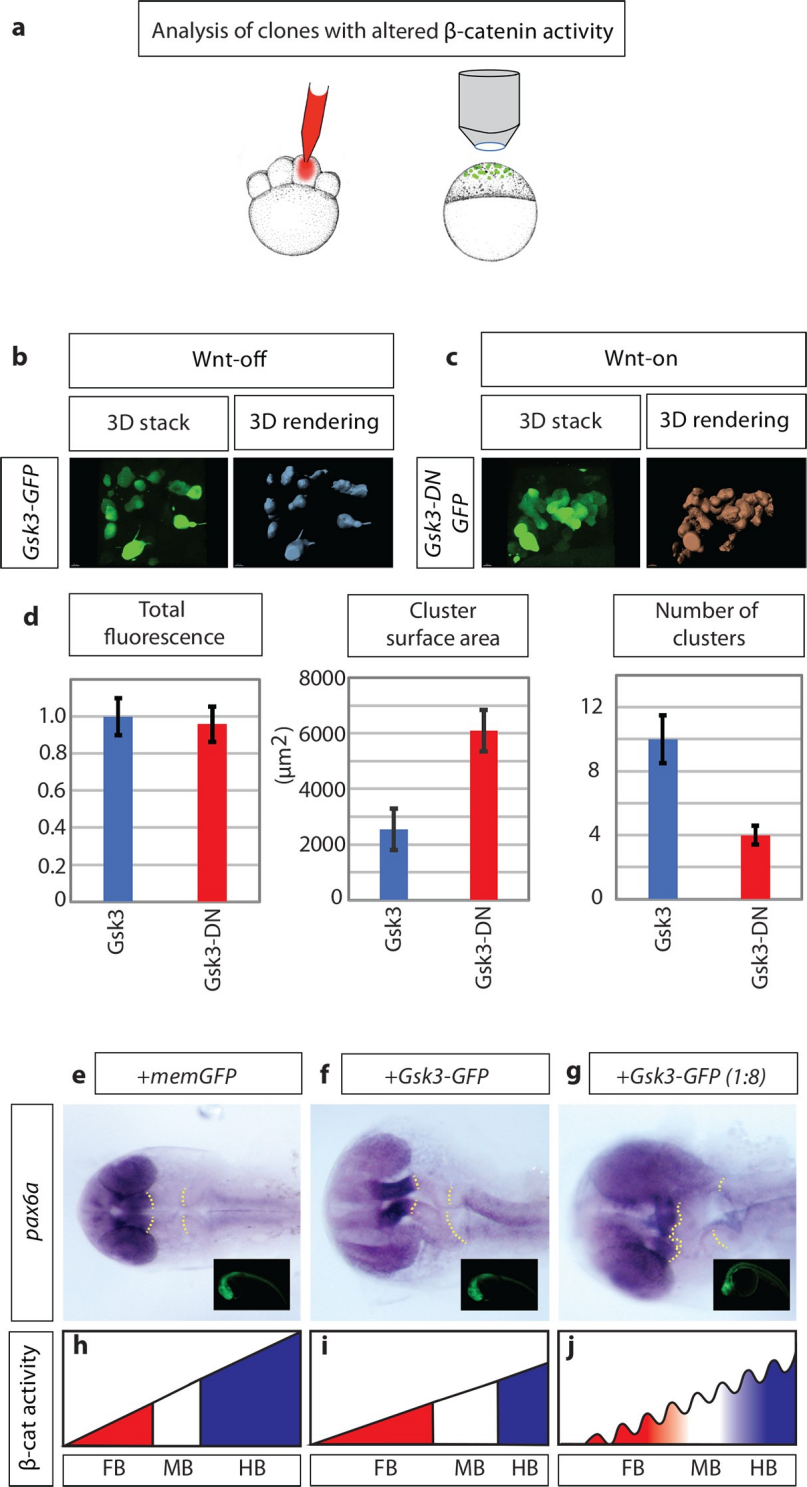
Wnt/β-catenin positive clones display increased adhesiveness and disrupt patterning. a) schematic illustration of the experimental procedure: 150ng mRNA injection of β-catenin effector Gsk3β into one cell at the eight blastomere stage. At sphere stage (prior induction of Wnt ligand expression), embryos were subjected to an image-based approach to analyse the distribution of cell clones in the animal tissue. b) expression of Gsk3β-GFP (Wnt-OFF) leads to a dispersed clone at the sphere stage. c) However, expression of dominant-negative Gsk3β-GFP (Gsk3β-DN-GFP; Wnt-ON) leads to clustering of the clonal cells. d) Gsk3β-DN expressing cells (Wnt-ON) show large clusters: demonstrated by an increased cluster surface and a reduced number of total clusters/cells compare to cells expressing WT Gsk3β (Wnt-OFF). The expression levels of Gsk3β and Gsk3β-DN are kept at a similar level shown by comparable total GFP-fluorescence in the clones. e)-g), embryos were injected with the indicated constructs and subjected to *in situ* hybridization against pax6a at 26hpf. The yellow dotted line illustrates the position of the neural plate boundaries. Insets show expression of the GFP tagged constructs prior fixation. h)-j), schematics illustrate the posterior shift of the borders after the reduction of β-catenin activity in the neural plate and the disruption of the boundaries after clonal decrease of β-catenin activity.

After degradation of maternally provided β-catenin and before the onset of endogenous Wnt expression, the zebrafish embryo comprises mostly cells with low Wnt/β-catenin activity (Wnt-off state). In addition, the embryonic tissue displays substantial cell rearrangements due to cell migration, proliferation and the onset of embryo-specific tissue rearrangements, e.g. epiboly movement. Therefore, we use this early embryonic tissue to perform a cell sorting assay. To this end, we generate cell clones with low Wnt/β-catenin level (Wnt-off) by over-expressing the key enzyme responsible for Wnt/β-catenin destruction, the glycogen synthase kinase-3β (Gsk3β) by micro-injection of its mRNA into one blastomere at the 16-cell stage. At 4 hpf, we find dispersed Wnt-off cells in the embryo (Fig 2).

In the next experiment, we express a dominant-negative form of Gsk3β, which lacks the kinase domain. This construct blocks β-catenin degradation (Wnt-on) and is used to enhance intrinsic Wnt activity in a ligand-independent and cell-autonomous way. We find that these Wnt-on cells with high β-catenin level form clusters (Fig 2). We quantify the results by calculating the average surface area of the clusters and find that Wnt-on cells form clusters that are three times larger compared to Wnt-off cells in a Wnt-off tissue. Our data suggest that cells may sort depending on their Wnt/β-catenin activity status.

Next, we addressed the question if altering β-catenin levels may disrupt border formation and thus, neural plate patterning. Therefore, we overexpressed Gsk3β -GFP to decrease β-catenin levels during early zebrafish development. At 26hpf, we fixed the embryos and performed an *in-situ* hybridization against the neural plate patterning marker *pax6a* (Fig 2). We find that overexpression of Gsk3β leads to an expansion of the forebrain primordium (FBP) including the eye field and midbrain primordium (MBP) at the expense of the hindbrain primordium (HBP) (Fig 2F; (n = 8/10) compared to embryos injected with the same amount of control mRNA (Fig 2E, n = 10/10). This finding is following the observation that reducing β-catenin level leads to an anteriorization of the neural plate (reviewed in [25]). The boundary between the forebrain and the midbrain primordium shifts towards the posterior; however, we observed no alteration of the sharpness of the border. Next, we overexpressed Gsk3β in one out of four blastomeres to generate a mosaic distribution of cells with a very low level of β-catenin (Fig 2G). Also, in these embryos, we find that the forebrain territory, including the eye vesicles, are increased. In parallel, we find that the boundary between forebrain and midbrain becomes fuzzy (n = 7/10). We, therefore, conclude that locally different levels of β-catenin in boundary cells—regulated by Gsk3β activity—can disrupt robust boundary formation (Fig 2H–2J).

### Directed migration in simulations

**Fig 3:**
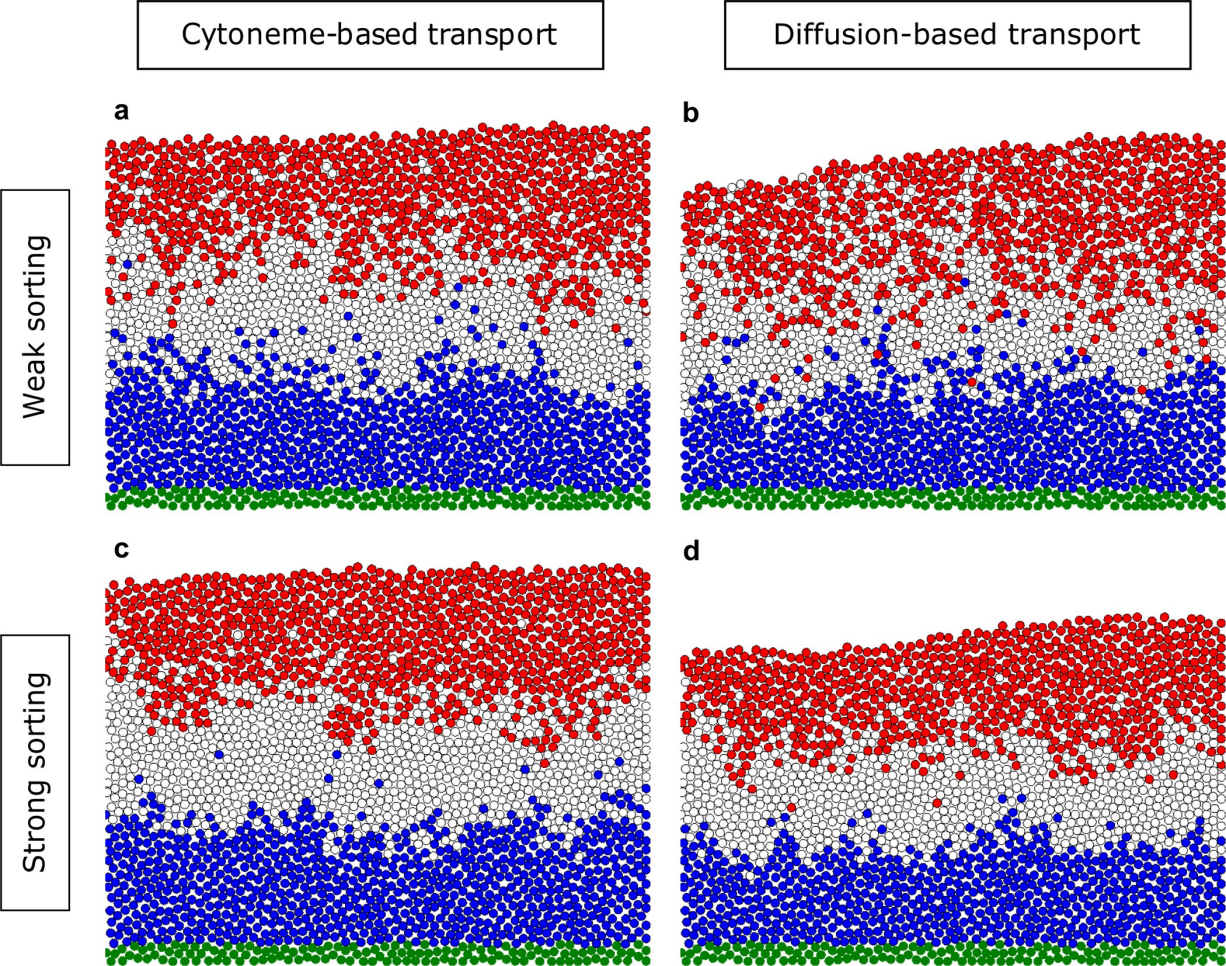
Boundary sharpening through directed migration. Distributions of the final cell fate at t = t_TRS_ = 180 min in the tissue for different parameters of the cell dynamics and different transport mechanisms. Directed migration with the sorting parameter *p*_DirMig_ = 0.02 is shown in **a)** and **b)**, and with *p*_DirMig_ = 0.2 in **c)** and **d)**. Cell fates and coloring are as in Fig 1.

Based on the experimentally observed nearest-neighbor cell swapping and β-catenin concentration-dependent cell sorting, we include directed migration in our model. To this end, we introduce directed nearest neighbor swapping in relation to the gradient with the sorting parameter *p*_DirMig_ (see methods). Based on a recent model for boundary sharpening [52], we test a range of values for *p*_DirMig_ ε [0…0.3] and display examples for weak sorting (*p*_DirMig_ = 0.02, cf. Fig 3A and 3B) and strong sorting (*p*_DirMig_ = 0.2, cf. Fig 3C and 3D). Notably, we find that a directed migration already even for weak sorting leads to drastic improvement in tissue patterning for both cytoneme-mediated and diffusive transport mechanisms. Increasing the sorting parameter by a factor of 10 leads to faster but qualitatively equivalent formation of well-defined borders (cf. Table 1). However, in both, the diffusion-based and in the cytoneme-based model, we find isolated cells located in the regions of dissimilar fates. This is to be expected from nearest neighbor interactions, as isolated cells behave diffusely if surrounded by homogeneously different cells and it becomes exponentially unlikely to escape homogenous large stretches of cells with different Wnt content.

**Table 1:** Cell fate environments. Shown are the percentages of cell fates that are surrounded by cells with mostly identical cell fates (>75%; “homogenous tissue”), mixed fates (25–75%; “borders”) or other fates (<25%, “isolated cells”) for the different simulations in Figs 1, 4 and 5. Considered are for each cell the 5 closest other cells as environment determined at t = 180 min.

	Fig 2		Fig 4				Fig 5	
			Weak Sorting	Strong Sorting	Weak Sorting	Strong Sorting	Apoptosis, weak sorting	No Apoptosis, weak sorting
	Cytoneme	Diffusion	Cytoneme	Cytoneme	Diffusion	Diffusion	Cytoneme	Cytoneme
**>75%**	32.6	50.9	82.0	87.1	74.3	86.4	80.8	76.1
**25–75%**	54.8	39.6	13.8	10.1	20.7	11.4	16.0	19.1
**<25%**	12.7	10.1	4.1	2.8	5.0	2.2	3.2	4.8

Overall, we find that both transport mechanisms combined with directed sorting lead to the formation of reproducible, robust boundaries, coinciding with the experimentally observed clear distinction between the different parts of the tissue. As the effect of the strong and rather un-physiological sorting parameter is not substantially bigger than the effect of the weak sorting parameter, subsequently we use *p*_DirMig_ = 0.02 (“weak sorting”) in the remainder of the manuscript.

### Induced apoptosis

**Fig 4:**
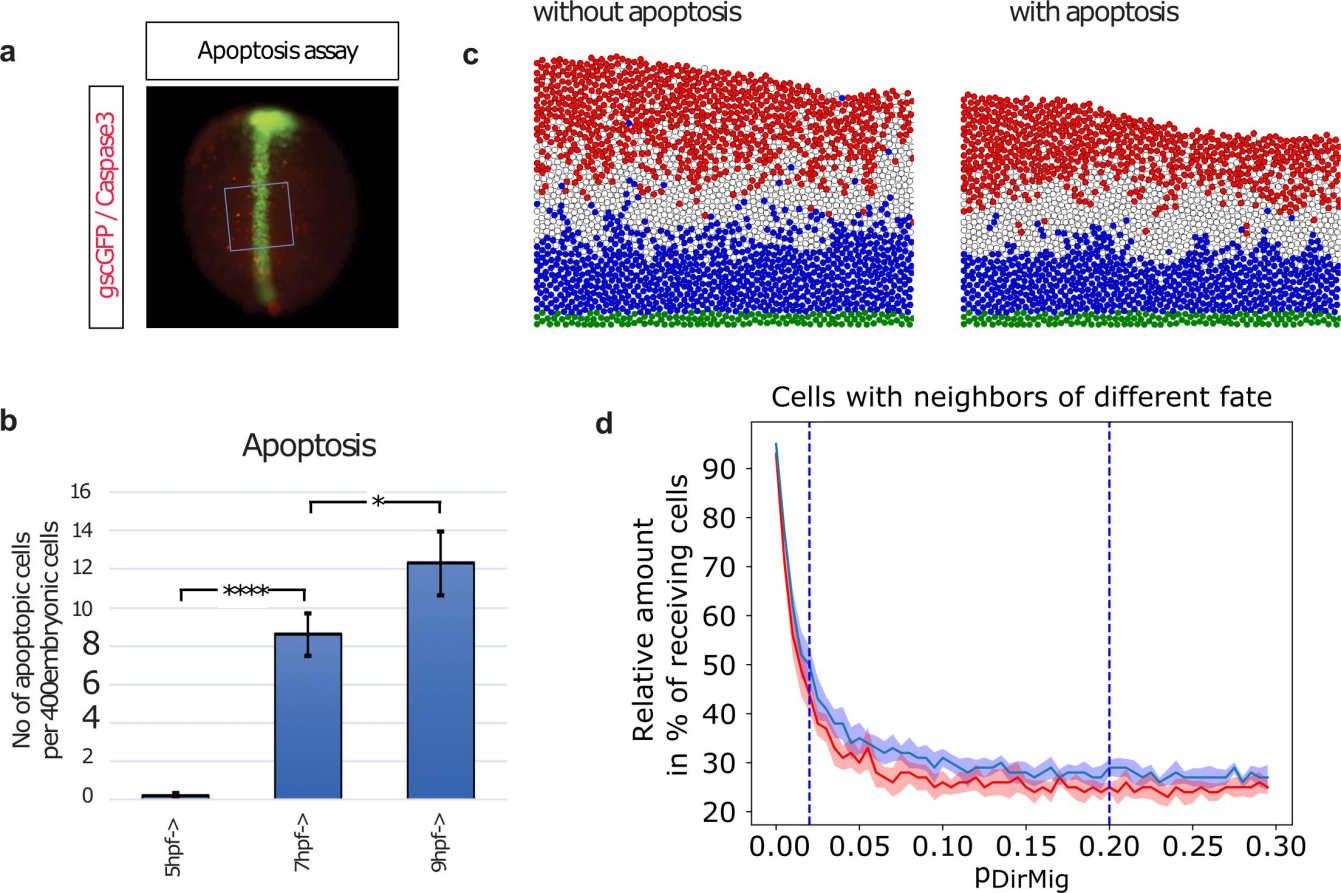
Apoptosis further improves neural plate patterning. To determine the number of apoptotic cells in the developing embryo, immunohistochemical staining against Caspase3 is performed at 5 hpf, 7 hpf, and 9 hpf. **a)**, After antibody staining, 10 areas of ca 20 by 20 cells are placed randomly over the dorsal hemisphere of the embryos (marked by *gsc*-GFP expression) and within these squares of ca 400 cells, the number of apoptotic cells is counted. **b)** At 5 hpf, no apoptotic cells are detected. At 7 hpf 2% and at 9 hpf 3% apoptotic cells are observed. **c)** Induced apoptosis of a similar relative number of cells as experimentally determined are implemented into our simulations. The simulation of cytoneme based Wnt-transport with weak sorting and no induced apoptosis (left) is compared to the simulation with additionally enabled induced apoptosis (right) at t = t_TRS_ = 180 min. Cell fates and coloring are as in Fig 1. **d)** Increasing the sorting parameter *p*_DirMig_ during the directed transport simulations leads to decreased mixing of cell fates (red with, blue without apoptosis). Even large values for p_DirMig_, however, cannot remove truly isolated cells (cf. c)). Adding limited apoptosis (130 cells over entire simulation) can eliminate these cells and leads to an additional improvement. Displayed is the development after *t* = 180 min averaged over 10 simulations for each value of *p*_DirMig_, with *p*_DirMig_ = 0.02 and *p*_DirMig_ = 0.2 (cf. Fig 4) highlighted.

Our simulations suggest that cell sorting is only effective at sharpening narrow regions between segments. If individual cells are located within a region with which they do not share the same fate and several rows away from the boundary zone, the local nature of contact-mediated cell-cell interactions cannot provide sufficient information to guide cells to the correct compartment. In several developmental contexts, apoptosis has been suggested to play an essential role in tissue remodeling during development [53, 54]. Indeed, cells with a strongly different Wnt/β-catenin activity compared to their neighbor cells undergo apoptosis by activating Smad signalling and reactive oxygen species. Therefore, we ask if programmed apoptosis could also provide a mechanism to reduce the number of outliers in the developing neural plate. Indeed, we experimentally observe a significant increase in the percentage of apoptotic cells observed during early zebrafish development at 5 hpf (not detectable), 7 hpf (2%) and 9 hpf (3%; Fig 4A and 4B). This increase at the stage of pattern refinement could point towards an induced cell death to aid patterning.

Based on these observations, we implement a mechanism for Wnt controlled apoptosis into our model. Here, apoptosis of a cell is potentially induced when the Wnt concentration of a single cell strongly differs from of all its surrounding neighbors. Apoptosis is only induced in a small number of cells (130 cells ~3.7% of all cells). After implementation, we find a further improvement of pattern formation (cf. Fig 4C, Table 1). In particular when introducing both directed migration and apoptosis the number of isolated cells is strongly reduced. Apoptosis without another sorting mechanism leads to no significant improvements of patterning as only few cells are affected (not displayed). Through the intrinsic stochasticity of the cytoneme based transport the stochasticity and the outliers are enhanced in the pattern. Therefore, the effect of apoptosis is mainly visible in those simulations. For diffusion-based transport the intrinsic stochasticity is lower through the long range of diffusion, therefor the impact of apoptosis is much less significant (cf. S7 Fig).

An alternative to apoptosis which might improve patterning would be “community decisions” of the cell fate. Community decisions prevent the formation of isolated cells by introducing the possibility of cellular trans-differentiation. This requires cells to sense both their own as well as the Wnt/β-catenin level of the neighboring cells and adopt their fate accordingly. We tested such a regime via short-ranged collective fate decisions (e.g. change of your own fate if majority of neighbors adopt a different fate). This, however, lead to the emergence of non-contiguous brain regions (cf. S4 Fig). Such neural development is not physiological, and we hence do not further explore this mechanism in our model.

Overall, we conclude that cell-specific apoptosis can reduce the prevalence of cells in the ‘incorrect’ regions which are far from pattern boundary regions. In turn, this leads to enhanced patterning across the neural plate.

### Timing of pre-patterning

So far, in our aim to establish stable tissue patterning via a concentration gradient in rapidly growing tissue we have observed that stochasticity effects lead to noisy spatial patterns. Neither diffusion nor cytoneme-based transport are able to generate sharp boundaries between distinct brain regions, nor are they able to eliminate isolated cells in brain regions with different fates. Our iterative approach of computational modeling and *in vivo* experiments suggests that cell sorting and apoptosis may operate in parallel to promote the formation and maintenance of a spatial pattern with sharp boundaries.

Surprisingly, there is no major difference between diffusion or a cytoneme-based trafficking mechanism with respect to the final spatial pattern. To this end, we ask if there is a temporal difference in inducing this pattern. This is a further important criterion as neural plate patterning is achieved within a few hours during early gastrulation [18]. Since it is not known at which time exactly cells adapt their fate, we investigate which time would provide the best conditions for fate acquisition followed a stable development of the pattern.

**Fig 5:**
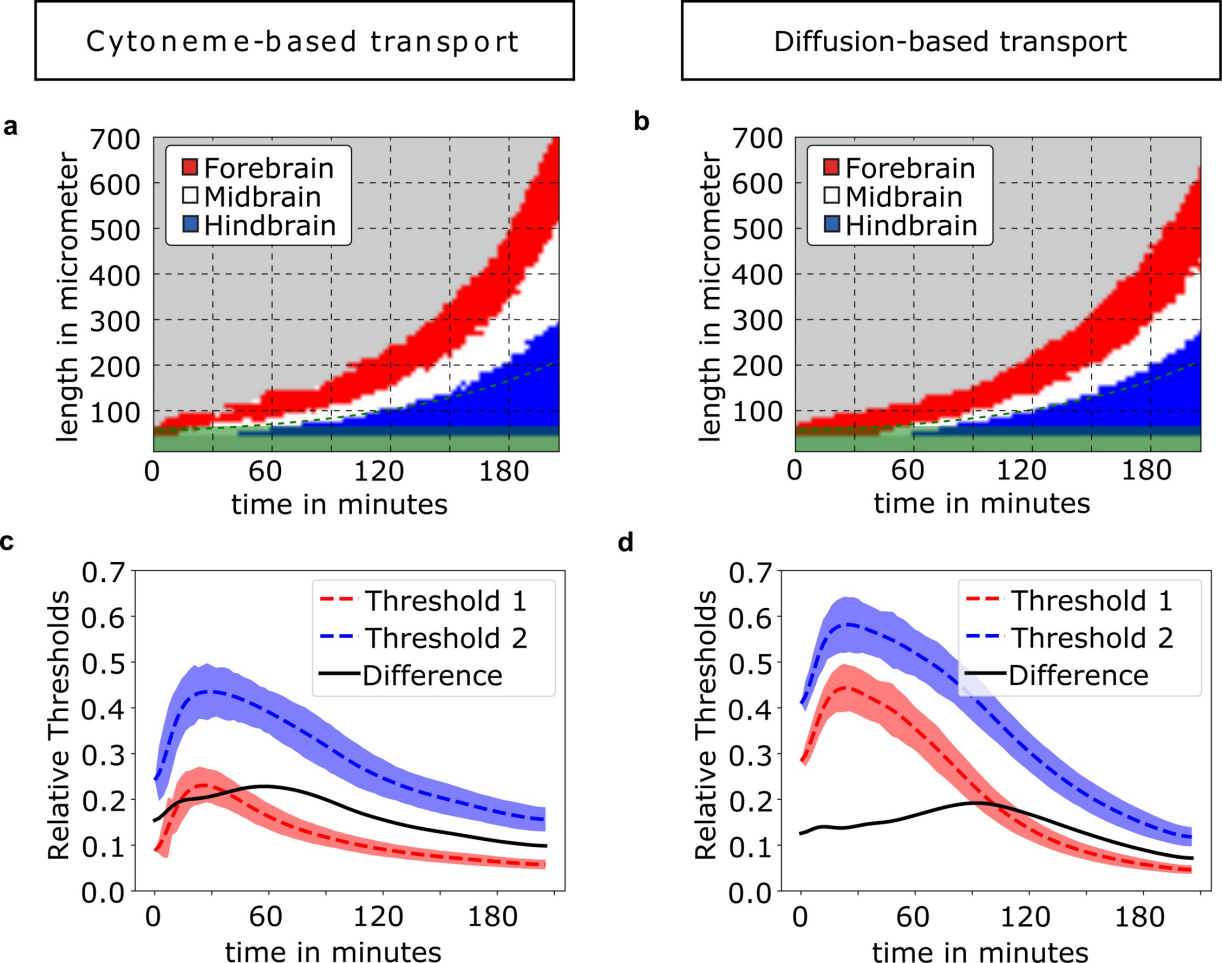
Timing of the pre-patterning for cytoneme-based and diffusion-based transport. In **a)** and **b)** the development of the pattern boundaries between brain areas during the tissue growth is shown. Colors indicate the respective brain primordium. The thresholds are set to split the tissue into thirds by number at t_TRS_ = 90 min. The green area indicates the layers of the morphogen producing cells. **c)** and **d)** show the thresholds for splitting the total number of cells into thirds at each respective point in time. The dashed lines are the averages of 100 simulations with the transparent regions showing the standard deviation σ. The lower threshold (red dashed line) separates forebrain and midbrain, the upper threshold (blue dashed line) separates midbrain and hindbrain. The difference between both thresholds (black line) indicates the distinctness between the thresholds separating cell fates.

To investigate the dynamics of the pattern formation, we analyze boundary formation in the simulations over time (cf. Fig 5). We observe a significant difference between the two transport modes: a cytoneme-based ligand mechanism achieves adequate thresholding earlier compared to diffusion-mediated transport (cf. Fig 5A and 5B). A quantitative description of this process can be achieved by observing the thresholds for separating the tissue into three areas and their development over time (Fig 5C and 5D). The acquisition of a definitive fate at the single cell level is challenging, especially when exposed to shallow single morphogen gradients. A well-defined difference of thresholds facilitates that choice through an easier derivation of the fate from the local Wnt concentration. The difference between both thresholds indicates the distinctiveness between different cell fates, with high differences being desirable. In comparison to the diffusion-based transport, we find that the cytonemal transport allows for an enhanced distinction over the entire simulated time and especially at early times between 20 and 60 minutes. These results suggest that formation of the neural plate involves pre-patterning with subsequent dynamic scaling of the neural plate primordium to achieve the desired dimensions.

**Fig 6:**
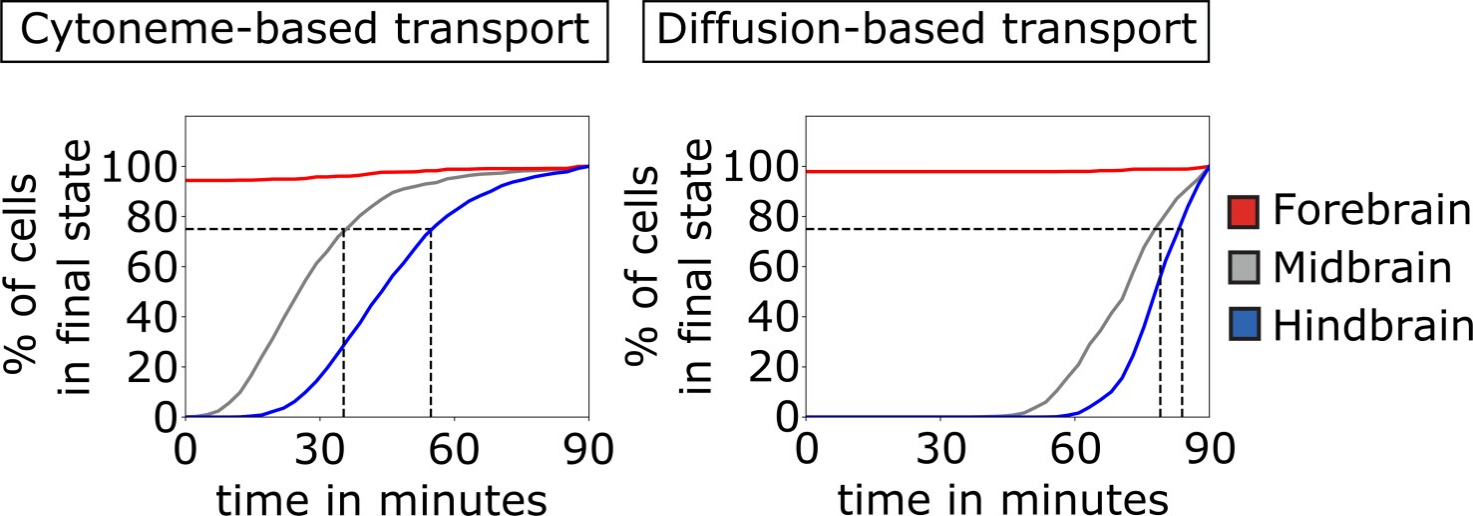
Quantification of pre-patterning. Comparison of the establishment of the three brain primordia based on cytoneme and diffusion-based transport. The percentage of cells that reach sufficient morphogen concentration to adopt their final cell fate (which is in this case determined by thresholding at t_TRS_ = 90 min so that the tissue is split into thirds by cell numbers) is plotted over time. This is shown for forebrain (red solid line), midbrain (gray solid line), and hindbrain (blue solid line). The gray dashed lines mark the points in time where 75% of the midbrain and hindbrain cells adopted their final fate, respectively. After about 45 min, the majority of cells (75%) acquire their final fate if the ligand is transported on cytonemes. A diffusion-based distribution requires considerably longer time (about 80 min) to determine the cellular fate in the target tissue. The values are averaged over 100 simulations.

To quantitatively describe these early patterning dynamics, we trace the fate acquisition of all cells from the start of the simulation until *t* = 90 minutes (cf. Fig 6). The final fate of a cell is assumed to be set at t_TRS_ = 90 minutes. From there each cell is traced back in time and the time is determined at which its Wnt concentration is sufficient for its final fate. With the cytoneme-based transport mechanism, more cells adapted their final fate and hence the tissue established the proper proportions of the pattern much earlier in development than with the diffusion-based transport. For instance, 75% of all cells adopt their final fate after 55 minutes with the cytoneme-based transport, whereas the diffusive transport is almost two times slower in reaching this threshold (t = 80minutes), when choosing the diffusion constant to be D = 0.1 μm^2^/s similar to the measured value of Wnt3-GFP [55]. Even for a ligand which diffuses orders of a magnitude faster patterning occurs at a similar time point or later (cf. S6 Fig). We conclude that there is a substantial difference regarding the timing between a cytoneme-based transport mechanism and a diffusion-based transport. We further find that the thresholds of different regions are more pronounced with cytoneme-mediated transports (cf. Fig 5C and 5D), which increases the robustness of patterning.

**Fig 7:**
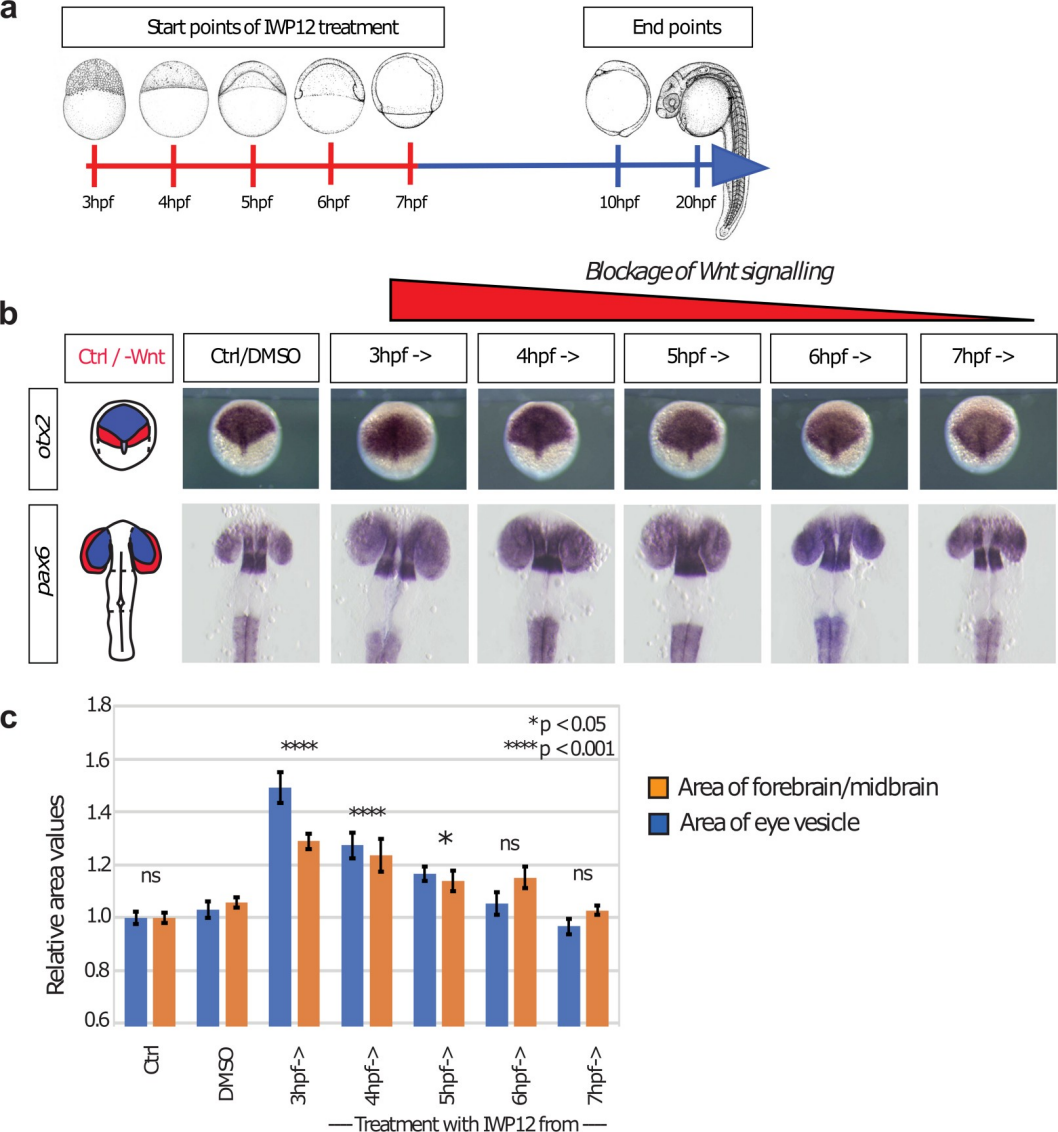
Wnt/β-catenin signaling influences anteroposterior patterning of the neural plate during early gastrulation. To determine the temporal function Wnt/β-signaling on patterning, zebrafish embryos are treated with 5 μM of the Porcupine inhibitor IWP12 to block Wnt secretion. **a)** Treatment started at different time points (3–7 hpf) until 10 hpf. At 10 hpf and 20 hpf, embryos are fixed and subjected to ISH against *otx2* and *pax6a*, respectively. **b)** Embryos treated from 3 hpf and 4 hpf to 10 hpf showed a significant increase of the forebrain and midbrain tissue at 10 hpf and an enlarged area of the eye vesicle at 20 hpf. **c)** Embryos treated after this time point showed only marginal changes in patterning, suggesting that Wnt signaling is required between 3–5 hpf to pattern the neural plate.

Our simulations suggest that cytoneme-mediated trafficking provides an advantage if there is a requirement for rapid adoption of final cell fate, e.g. in a quickly expanding tissue. Therefore, we hypothesize that such a mechanism could be preferred for the patterning of the neural plate in zebrafish. We experimentally test this hypothesis by blockage of Wnt production at specific time windows during early embryonic development (Fig 7). To this end, we treat the embryos with IWR12—an inhibitor of Porcupine, which is essential for lipidation of Wnt proteins (Fig 7A). Treatment with IWR12 leads to the production of un-lipidated Wnt proteins, which are blocked from secretion and quickly degrade [56]. All Wnt molecules produced prior to treatment are still secreted and transported and thus have the possibility to signal. To analyze an alteration in AP patterning of the neural plate, we measure the area of the forebrain/midbrain marked by *otx2* expression at 10 hpf and the area of the *pax6a* positive eye vesicles at 24 hpf. We find that blockage of Wnt production at the onset of Wnt expression between 3 hpf– 4 hpf is most effective and increases the size of the forebrain/midbrain area of about 43% and the size of the eye vesicle of about 24%, respectively (Fig 7B and 7C). Later blockage after 4 hpf does not have strong effects. Therefore, Wnt/β-catenin signaling is most effective in setting a pattern within the first hour of Wnt8a expression.

Based on our simulation results and the experimental validation, we suggest that Wnt-mediated patterning of the neural plate is a fast process, which requires pre-patterning of the neural plate primordium by a cytoneme-based transport of the key morphogen with subsequent dynamic scaling including cell sorting and directed apoptosis. Experimentally we find that the fate of the cells and therefore the tissue prepattern is defined within the first hour of Wnt expression, in unison to that we independently found in our modeling studies that cytoneme-based transport provides significant advantages over diffusion-based transport at pattern formation in an expanding tissue and similar time ranges of one hour.

## Discussion

### Cytonemes as transport vehicle for Wnt morphogens

Spatial patterning during embryogenesis is orchestrated by morphogens that control tissue growth and differentiation at a distance from the morphogen-producing cells. Turing’s morphogen model [1] and Wolpert’s French flag model [3] dominate our understanding of tissue patterning and cell differentiation. The underlying assumption of simple diffusion of morphogens produced by source cells, which ultimately determines cell fate through the establishment of morphogen gradients, has been recently challenged due to the observation of other complex transport mechanisms [8]. Recently, a cytoneme-based transport mechanism has been linked to morphogen distribution [10]. Cytonemes are specialized filopodia that can, despite only delivering morphogens by short range directed transport, mediate long-distance signaling during development [9]. This directed transport offers a more controlled way of distributing morphogen and, therefore, might be more suitable for the rapid generation of a precise gradient during early development.

We find that the cytoneme-based transport of Wnt allows for fast gradient formation in the neural plate within minutes (Figs 5 and 6). Indeed, Wnt cytonemes have been shown to target the receiving cell in less than 10 min [22]. The gradient also requires correct signaling strength, which could be controlled by cytonemes through the frequency of contact with the recipient cells. The amount of ligand present may also influence signal activation, however, data that substantiate this assumption are sparse. We find that the Wnt gradient depends on the time during which the recipient cells are in reach of the cytonemes and the stability of the induced ligand–receptor complex. An establishment of a pre-pattern is a direct consequence of this transport mechanism.

### Dynamic scaling of a pattern

Besides ligand transport, a further critical issue is the maintenance of morphogen thresholds, in particular, in border regions, during growth. Tissue expansion and cell migration largely influence patterning in quickly forming embryonic tissues. Therefore, the absolute positions of boundaries must be flexibly but proportionally adopted. As these boundaries are set and maintained by a specific Wnt concentration threshold, the profile of these specific morphogen concentrations or the thresholds must be dynamically adjusted. Different models have been proposed to explain dynamic scaling of morphogen gradients in various systems. An intuitive explanation would be the adjustment of ligand production to tissue size or by a tissue size-dependent change in the turn-over rate as suggested for TGFβ signals [57]. Such a mechanism would imply that the tissue “knows” about its total size and adjusts its signal strength accordingly. However, in the case for TGFβ signaling, it has been questioned if it impacts on downstream events, such as cell proliferation [58].

In vertebrates, morphogens such as Sonic hedgehog (Shh) and Bone morphogenic proteins (BMP) form a dorsoventral gradient in the spinal cord [59]. Recent data has demonstrated that these morphogens determine cellular fate of spinal cord neurons and regulate the size of groups of neurons by domain-specific regulation of differentiation rate [60]. An alternative model suggests that linear diffusion can only account for morphogen gradients in differentially scaled species if the life-time of the morphogen is adjusted to the size of the field to be patterned, as suggested for the bicoid gradient in oocytes of related fly species [61]. Similarly, in the wing imaginal disc a further model has recently been proposed, in which pre-steady-state dynamics and ligand advection following linear diffusion would allow the establishment of the Decapentaplegic (Dpp) morphogen gradient [36]. In summary, these experiments and simulations suggest that gradients can be adapted to growing tissue by different mechanisms, thereby allowing dynamic scaling to tissues with different sizes. However, none of these descriptions consider a cytoneme-based transport.

With this study, we identify a mechanism to establish and maintain precise and functional patterns in dynamic tissues. Our simulations suggest that scaling of the Wnt morphogenetic gradient on an expanding neural plate is the result of a pre-patterning event followed by tissue expansion. This implies fast cytoneme-mediated ligand transport followed by a rather long half-life of the ligand-receptor complex—termed Wnt signalosome. Recent data in cell culture suggest that the Wnt signalosome influences gene transcription over 10h post formation [62]. This time window is in accordance with our proposed model. Ligand advection has been suggested as a crucial parameter for morphogenetic field formation by Dpp in the Drosophila wing [36]. However, our data suggest that—independent of the ligand concentration—advection of active Wnt signalosomes is crucial in Wnt signal maintenance [38]. We propose that after the pre-patterning phase, the distribution of Wnt signalosomes in the neural plate links the processes of gradient scaling and tissue growth.

### Translation of a morphogen gradient into a spatial pattern

Although we incorporate several improvements and refinements in our model, such as a complex propagation mechanism of the morphogen and acquisition of cell fates on the single cell level in 2D, this model also has limitations. We establish a simple and robust model which focuses on 2D mapping of epithelial-like tissue. The 3D aspect of the developing neural plate is realized by intercalation and implicit treatment of cell division via probabilities, rather than by explicit inclusion. Furthermore, our model is approximative in that it treats all cells as having fixed positions with identical sizes. Group motion of cell clusters and cell adhesion are likewise only implicitly modeled. The implication of Wnt content dependent apoptosis is hypothetical and direct experimental evidence for such a mechanism should be provided in the future. Also, the characteristics of the Wnt-dependent signaling cascades including its internal, Axin-dependent negative feedback regulation could be included in future versions of the model. The described extensions, however, would vastly increase model complexity as well as parameter space, requiring highly accurate and reliable experimental efforts to parametrize, outside the scope of the current study.

**Fig 8:**
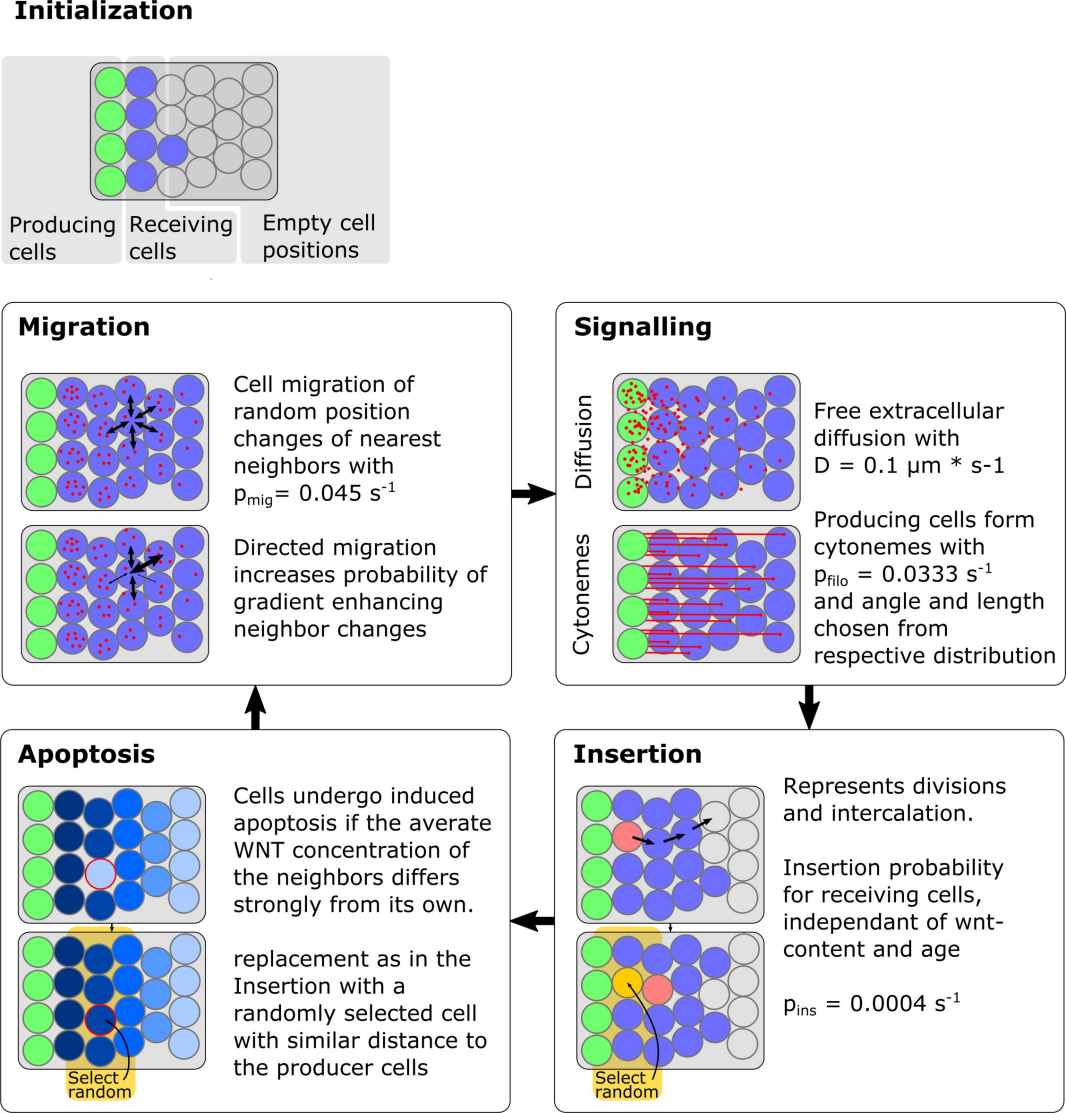
Overview over the simulation method and model parameters. The field in which the tissue is modeled consists of precomputed non-overlapping potential cell positions (“fixed irregular lattice sites”). To initialize a simulation one margin of the field is filled with non-dividing morphogen producing cells (here shown in green), as well as morphogen receiving cells (shown in blue) with no initial morphogen concentration. After initialization (up to) four processes are sequentially executed for one timestep. In the *signaling* step morphogen is deposited from the producing into the receiving cells, either by diffusive transport or by direct cell to cell contacts via cytonemes, also some morphogen decays here in the recipient cells. *Insertion* drives the tissue growth and consists of two causes which cause the same effect, namely cell division and intercalation from overlying and underlying cell sheets. Here, if an insertion occurs at one cell, a path to the nearest empty cell position is determined and all cells are subsequently moved along this path, the emerging empty spot is than filled by a cell with its properties chosen randomly from all cells with similar distance to the producing cells. Apoptosis can be induced if the Wnt concentration of a single cell differs strongly from its neighbors, this process is only activated in the last third of the simulation and only the 130 cells with the strongest discrepancy of Wnt contents to neighbors are removed and replaced similarly to insertion. *Migration* models nearest neighbor interactions by a random swapping of positions of nearest neighbor cells. Directed migration can be introduced if the swapping probabilities are changed so that gradient enhancing swaps become more likely.

In a straightforward implementation of morphogen transport in 2D with an expanding tissue domain, both transport mechanisms diffusion and cytoneme-based transport are able to establish stable 1D concentration gradients (Fig 1) [63]. These 1D graphs, however, hide the underlying strong intermingling of cell fates in a sheet of cells. Looking at the discrete 2D tissue representation, we do not observe sharp boundaries between neighboring tissue regions even to the point that regions are difficult to distinguish (cf Fig 1). Our model assumes that morphogen is deposited in the tissue by cytonemes which are assigned experimentally validated distributions of lengths and angles [22]. Based on the experimental evidence for cell migration [29] and Wnt concentration dependent cell clustering (Fig 2), we incorporate a Wnt concentration dependent directed cell sorting mechanism into our model framework (cf. Fig 8). Even weak directed migration of cells considerably improves the cohesiveness of patterning for both modes of transport (Fig 3). Following the formation of the Wnt morphogen gradient, we find that a sorting mechanism is essential for patterning and pattern maintenance to counteract the single-cell stochasticity that is introduced by the dynamical intercalation and expansion of the cell sheet. A cell-sorting based mechanism is crucial to establish patterning in a variety of other systems, including the Shh morphogenetic field, which specifies the cellular fate of spinal cord neurons in zebrafish [64].

Still, there remain individual isolated cells of different fate in otherwise contiguous tissue regions, which are not observed in experimental data. As a potential mechanism to address this, we experimentally investigate the possibility of apoptosis of isolated cells. Indeed, there is good evidence that Wnt signaling components such as sFRP2, Apc, Gsk3β, and β-catenin regulate apoptosis [65–67]. We hypothesize that the occurrence of induced apoptosis could be driven by differences in cellular fate or Wnt concentration compared to the neighboring cells. A mechanism how cells compare their fate or concentration to the neighboring cells has been suggested recently: cells with a lower b-catenin concentration receive a signal via cadherin stimulating apoptosis by activating Smad signalling and reactive oxygen species production. By introducing induced apoptosis to the model during the pattern refinement stage, we find an improvement of patterning cohesiveness and refinement (cf. Fig 4). In summary, we speculate that three consecutive steps are required to establish anteroposterior patterning in the neural plate: a graded cytoneme-based distribution of the Wnt morphogens, followed by a cell sorting process depending on the β-catenin activity levels, and finally by a selective apoptotic mechanism to eliminate isolated cells.

### Transport mode influences timing of patterning

Observing the final pattern shape, it appears that there is only a slight difference between diffusion-based and cytoneme-based transport mechanisms of morphogens in the ultimate generation of a morphogenetic field. By comparing the temporal emergence of tissue pattern, we find that cytoneme-based transport of morphogens leads to early establishment of sufficiently different concentrations to develop all three regions of the neural plate (cf. Figs 5 and 6). Diffusion-based transport also allows for the establishment of a pattern, but this takes significantly more time to establish the correct proportions of the tissue. This provides a considerable advantage of cytoneme-based transport, as we hypothesize that the early establishment of a definite cell fate by pre-patterning is more stable and less prone to errors. The main distinction between the modes of transport is visible within the first hour of Wnt expression, at the same time we experimentally identified Wnt signaling in the first hour to be most important for correct pattering (cf. Fig 7). Since cytoneme based transport provides advantages in pattern definition in the time that the pattering occurs in zebrafish neural plate development we hypothesize that cytonemes are a more reliable and therefore evolutionary more favorable patterning mechanism for fast pattern generation. The patterning mechanism we suggest here, is an early pre-patterning phase of the tissue with subsequent dynamic scaling and pattern refinement through Wnt concentration dependent cell sorting and apoptosis. In addition, cell-contact-based transport leads to more distinct concentration thresholds (cf. Fig 5), which makes the system less error-prone and gives rise to more distinct boundaries of regions.

In summary, there is a requirement for gradient scaling during embryogenesis, which is achieved by an early pre-patterning followed by a cell sorting mechanism. Pre-patterning by cytoneme-based morphogen transport and simultaneous growth of the receptive field have become recurring themes in morphogen-controlled systems. The here-described model, with an exemplar of neural plate formation in zebrafish, is of general use for pre-patterning and dynamic scaling in fast growing tissues, e.g. during embryogenesis.

## Supporting information

S1 Table Parameters used in the simulations. (XLSX)

S1 Fig Schematic overview/ Graphical abstract. During zebrafish gastrulation a pattern is introduced within the expanding tissue via a morphogen gradient. Two transport mechanisms of morphogen, namely diffusion and direct cell-cell contact via signaling filopodia are compared. In addition, fast expansion of the recipient tissue with enhanced tissue plasticity and controlled cell death are introduced to refine the pattern in the neural plate. Green indicates the Wnt protein producing cells at the embryonic margin. In turn, the neural plate responds to the Wnt morphogenetic gradient by adopting different cellular fates: High Wnt activity—hindbrain (blue), low Wnt activity—midbrain (white), and no Wnt activity—forebrain (red).
(TIF)

S2 Fig Angular distribution of filopodia. (Data from [22]).
(TIF)

S3 Fig Histogram of cell nearest neighbor lifetimes. The nearest neighbors are identified for each cell in every time step and the lifetime of neighborhood relationships is measured. The data reveilles a highly dynamic behavior and large contributions from very short lifetimes. Data from the lightsheet data set [47].
(TIF)

S4 Fig Community fate decision. In this scenario the fate of the individual cell does not only depend on its Wnt content but also on the fate its nearest neighbors have acquired. The fate of the cells is initialized solely by a threshold on the Wnt-concentration at t = 90 minutes. Subsequently, every 20 simulation sweeps, the fate of the cells is updated with the probabilities ß*p_wnt_ based on the Wnt-concentration and γ*p_nei_ based on the fate of the neighbors. The mechanism is sketched in a). b) shows a simulation run without the community fate decision enabled and c) shows a simulation run incorporating the mechanism. One can see a clustering of the individual cell fates, but rather the formation of patches than a stripe pattern. Besides the Wnt producing cells shown in green, the colors of the cells represent different cellular fates: forebrain fate is indicated in red, midbrain fate in white and hindbrain fate in blue.
(TIF)

S5 Fig Wnt-gradient. Simulation output of the Wnt-gradient at different time points. 100 simulations are run, depicted is the mean value (solid line) with the standard deviation (shaded area). The simulations are run for (upper) cytoneme based transport with directed migration enabled (p_DirMig_ = 0.02) and (lower) diffusion-based transport. The normalization is relative to the peak value after 180min in the respective simulation.
(TIF)

S6 Fig Comparison of different diffusion constants and boundary conditions. The simulations are performed with Diffusion constants D = 0.000001 μm^2^/s to D = 100 μm^2^/s (experimentally found values between 0.01 and 7 μm^2^/s [69, 70]). As well as a varying source cell Wnt concentration V_0_ ε [0.01, 100]. Neither varying the diffusion constant nor V_0_ leads to a significantly earlier possibility for prepatterning. Thresholds are set as in main text Fig 6.
(TIF)

S7 Fig Impact of apoptosis on diffusion-based transport. Top weak sorting (left without and right with apoptosis). Bottom medium sorting (left without and right with apoptosis). Apoptosis does not strongly impact the patterning for diffusion-based transport in our simulations.
(TIF)

S8 Fig Temporal development of pattern formation. Simulation snapshots of the emerging tissue and its pattern, depicting one exemplary simulation each from Figs 5 and 6. In the top six images diffusion-based transport is shown and in the bottom six images cytoneme based transport is shown. The earlier and more robust establishment of a stable three stripe pattern can be observed in the cytoneme based transport. The thresholds are set to split the tissue into thirds by number at t_TRS_ = 90 min.
(TIF)

S9 Fig Scheme of cell movements during cell division and directed migration. (TIF)
