# Supplementary figures and images for: Modeling of Wnt-mediated tissue patterning in vertebrate embryogenesis
Source: PLoS Comput Biol. 2020 Jun 24;16(6):e1007417. doi: 10.1371/journal.pcbi.1007417 (PMC7340325; doi:10.1371/journal.pcbi.1007417)

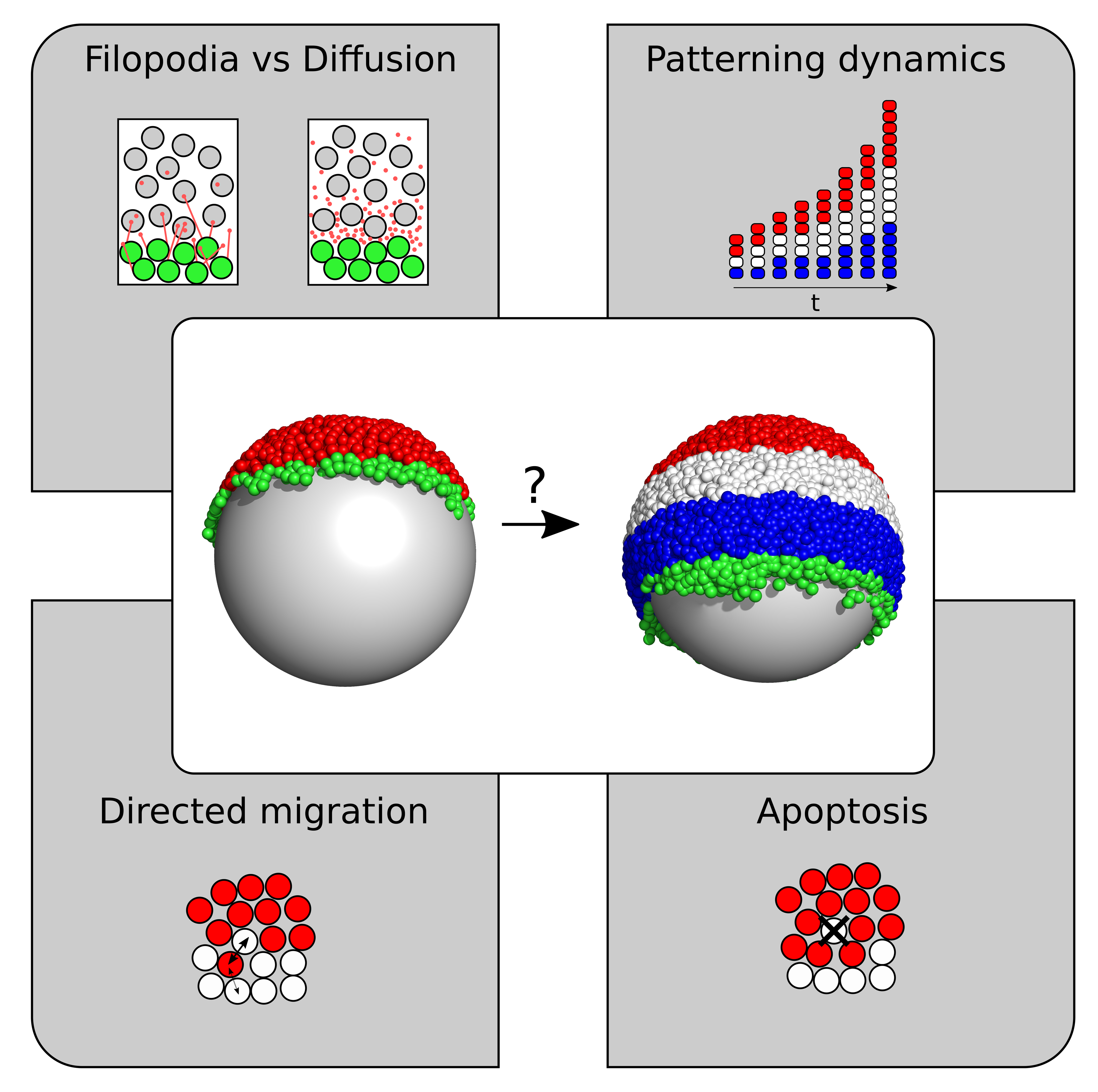

Supplement: S1 Fig — During zebrafish gastrulation a pattern is introduced within the expanding tissue via a morphogen gradient. Two transport mechanisms of morphogen, namely diffusion and direct cell-cell contact via signaling filopodia are compared. In addition, fast expansion of the recipient tissue with enhanced tissue plasticity and controlled cell death are introduced to refine the pattern in the neural plate. Green indicates the Wnt protein producing cells at the embryonic margin. In turn, the neural plate responds to the Wnt morphogenetic gradient by adopting different cellular fates: High Wnt activity—hindbrain (blue), low Wnt activity—midbrain (white), and no Wnt activity—forebrain (red). (TIF) [file pcbi.1007417.s002.tif]

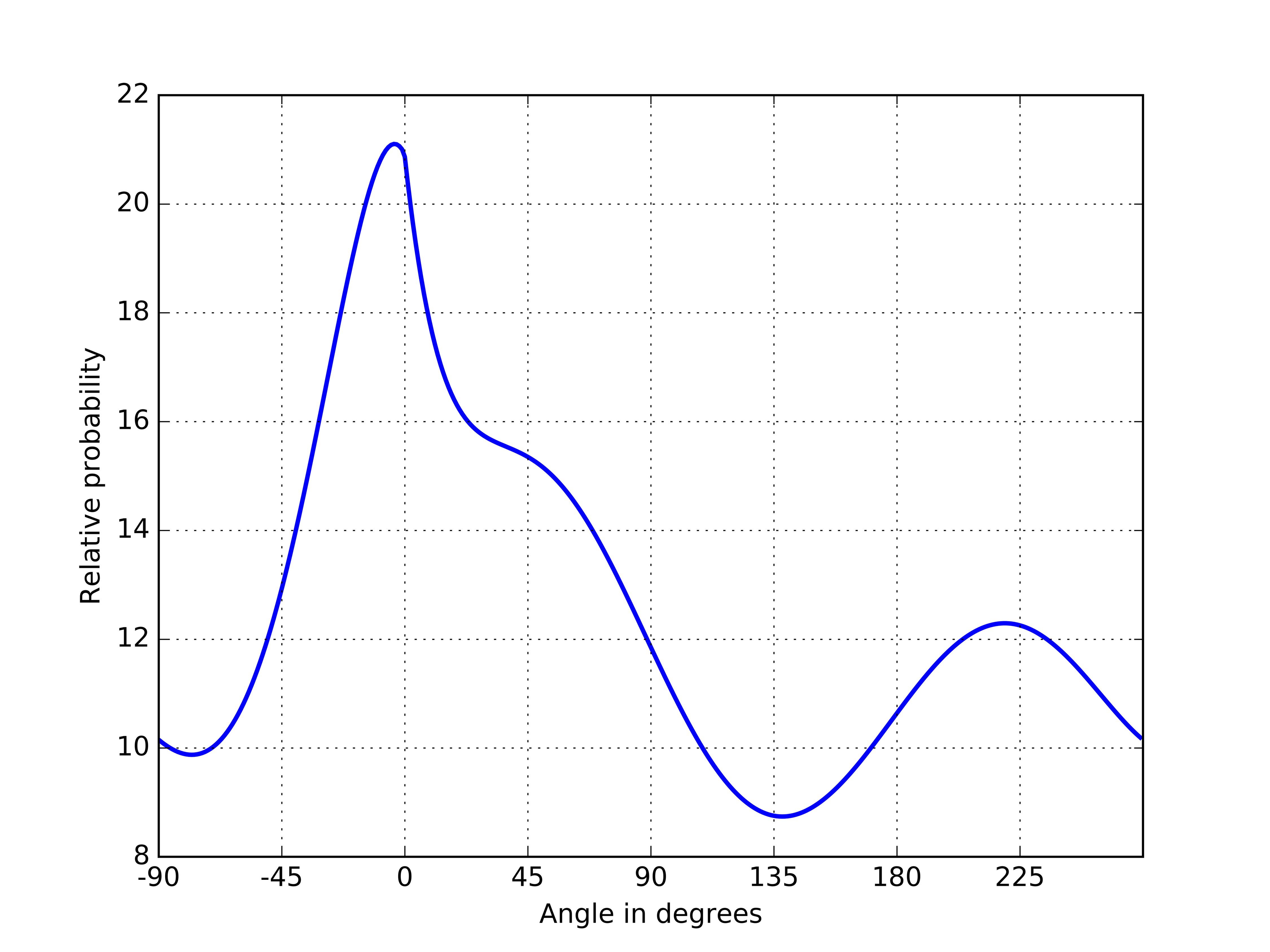

Supplement: S2 Fig — (Data from [22]). (TIF) [file pcbi.1007417.s003.tif]

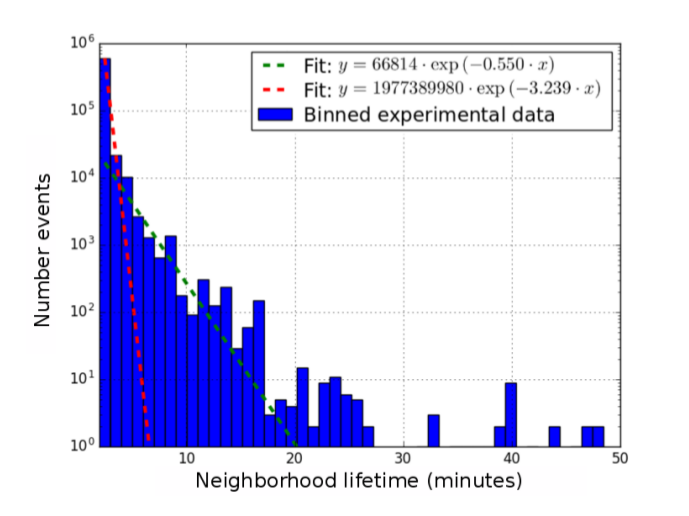

Supplement: S3 Fig — The nearest neighbors are identified for each cell in every time step and the lifetime of neighborhood relationships is measured. The data reveilles a highly dynamic behavior and large contributions from very short lifetimes. Data from the lightsheet data set [47]. (TIF) [file pcbi.1007417.s004.tif]

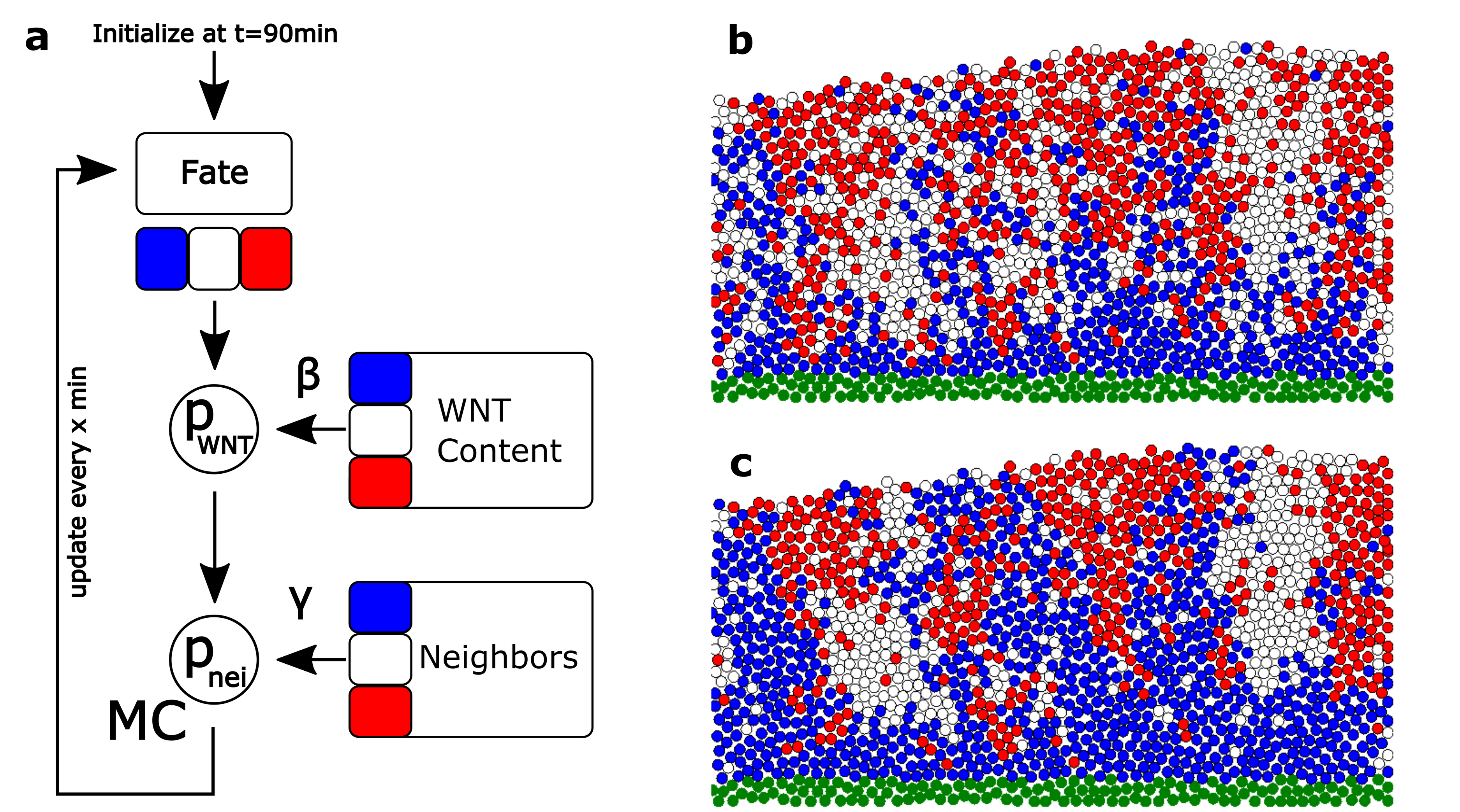

Supplement: S4 Fig — In this scenario the fate of the individual cell does not only depend on its Wnt content but also on the fate its nearest neighbors have acquired. The fate of the cells is initialized solely by a threshold on the Wnt-concentration at t = 90 minutes. Subsequently, every 20 simulation sweeps, the fate of the cells is updated with the probabilities ß*pwnt based on the Wnt-concentration and γ*pnei based on the fate of the neighbors. The mechanism is sketched in a). b) shows a simulation run without the community fate decision enabled and c) shows a simulation run incorporating the mechanism. One can see a clustering of the individual cell fates, but rather the formation of patches than a stripe pattern. Besides the Wnt producing cells shown in green, the colors of the cells represent different cellular fates: forebrain fate is indicated in red, midbrain fate in white and hindbrain fate in blue. (TIF) [file pcbi.1007417.s005.tif]

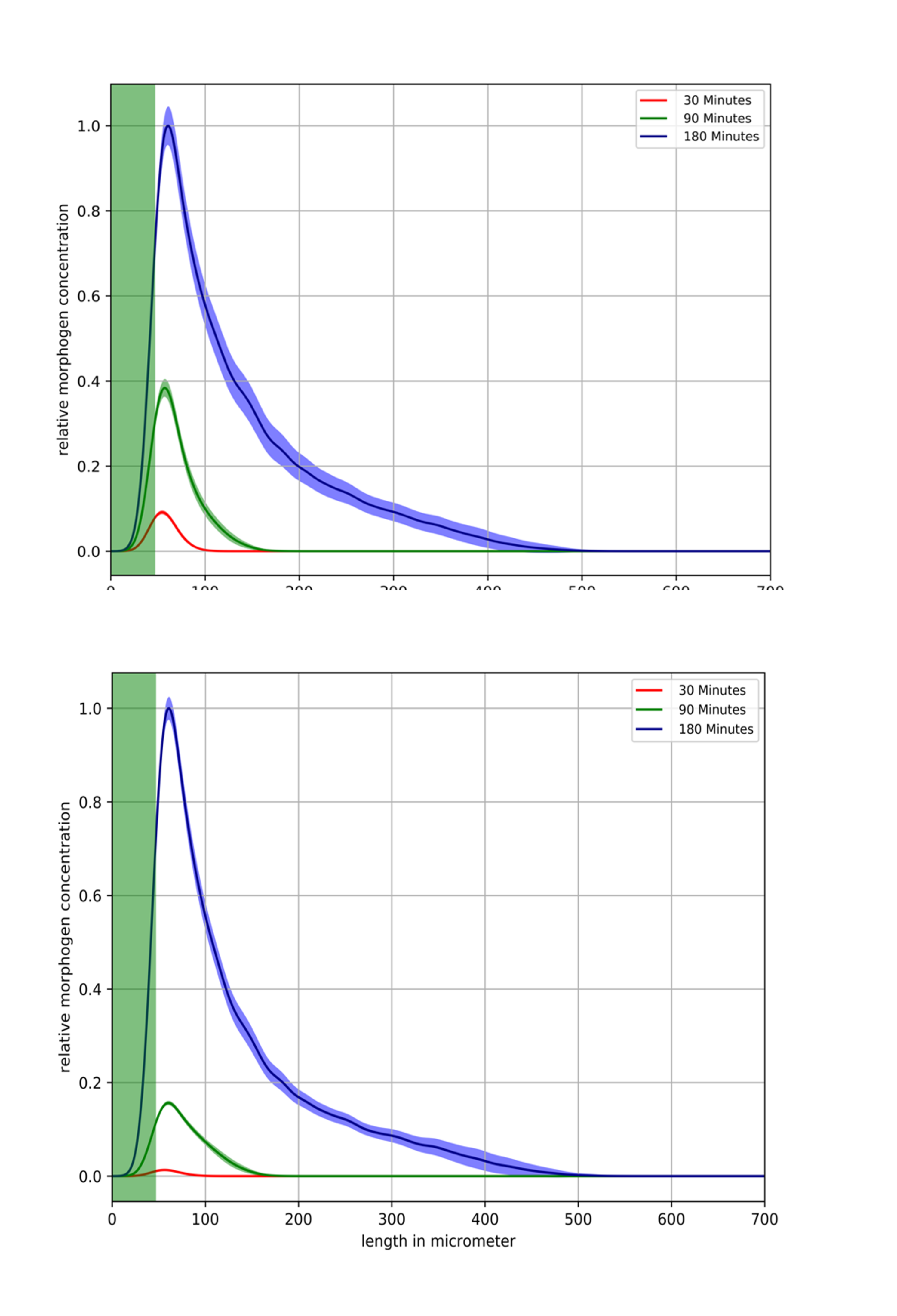

Supplement: S5 Fig — Simulation output of the Wnt-gradient at different time points. 100 simulations are run, depicted is the mean value (solid line) with the standard deviation (shaded area). The simulations are run for (upper) cytoneme based transport with directed migration enabled (pDirMig = 0.02) and (lower) diffusion-based transport. The normalization is relative to the peak value after 180min in the respective simulation. (TIF) [file pcbi.1007417.s006.tif]

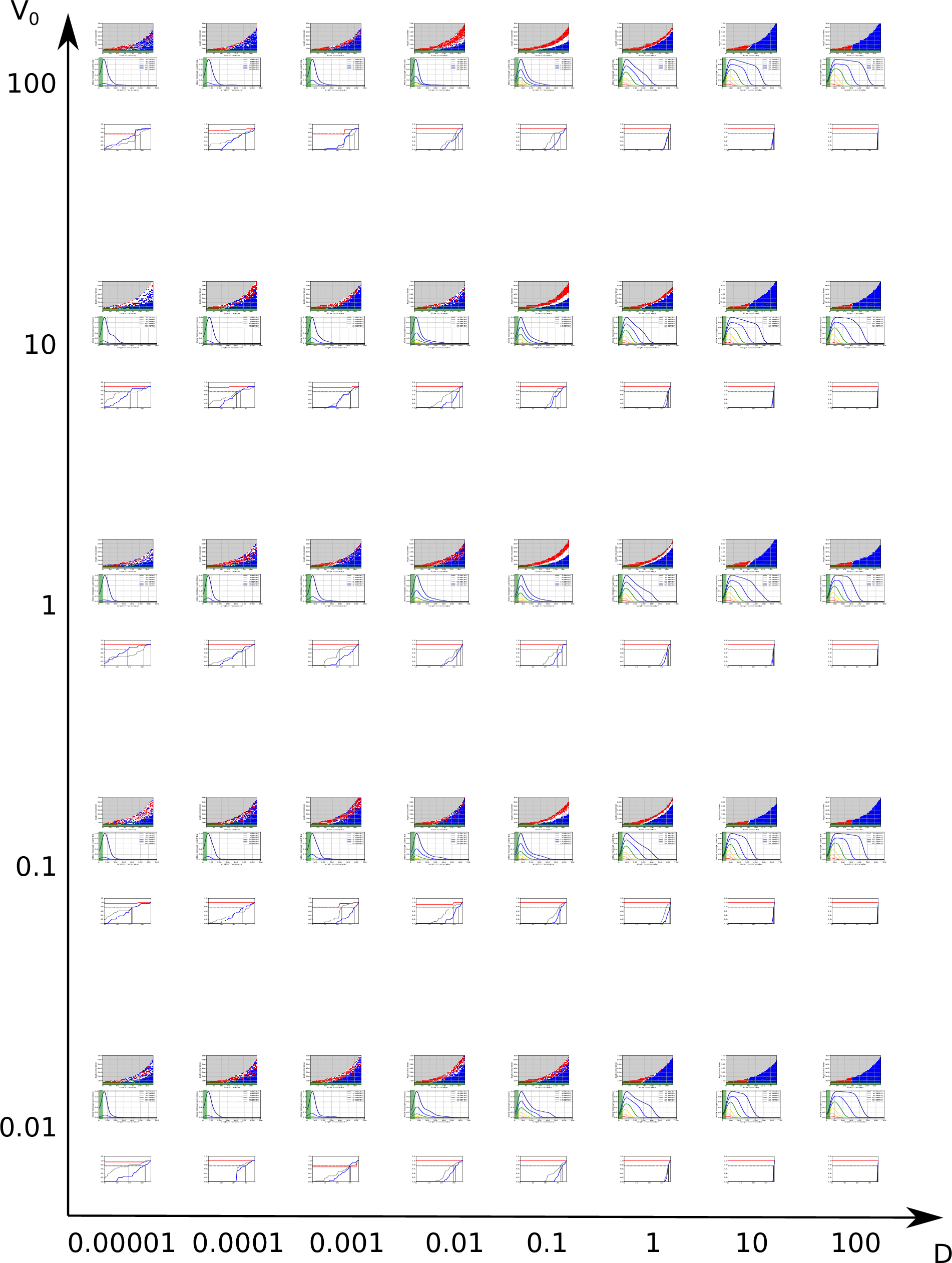

Supplement: S6 Fig — The simulations are performed with Diffusion constants D = 0.000001 μm2/s to D = 100 μm2/s (experimentally found values between 0.01 and 7 μm2/s [69, 70]). As well as a varying source cell Wnt concentration V0 ε [0.01, 100]. Neither varying the diffusion constant nor V0 leads to a significantly earlier possibility for prepatterning. Thresholds are set as in main text Fig 6. (TIF) [file pcbi.1007417.s007.tif]

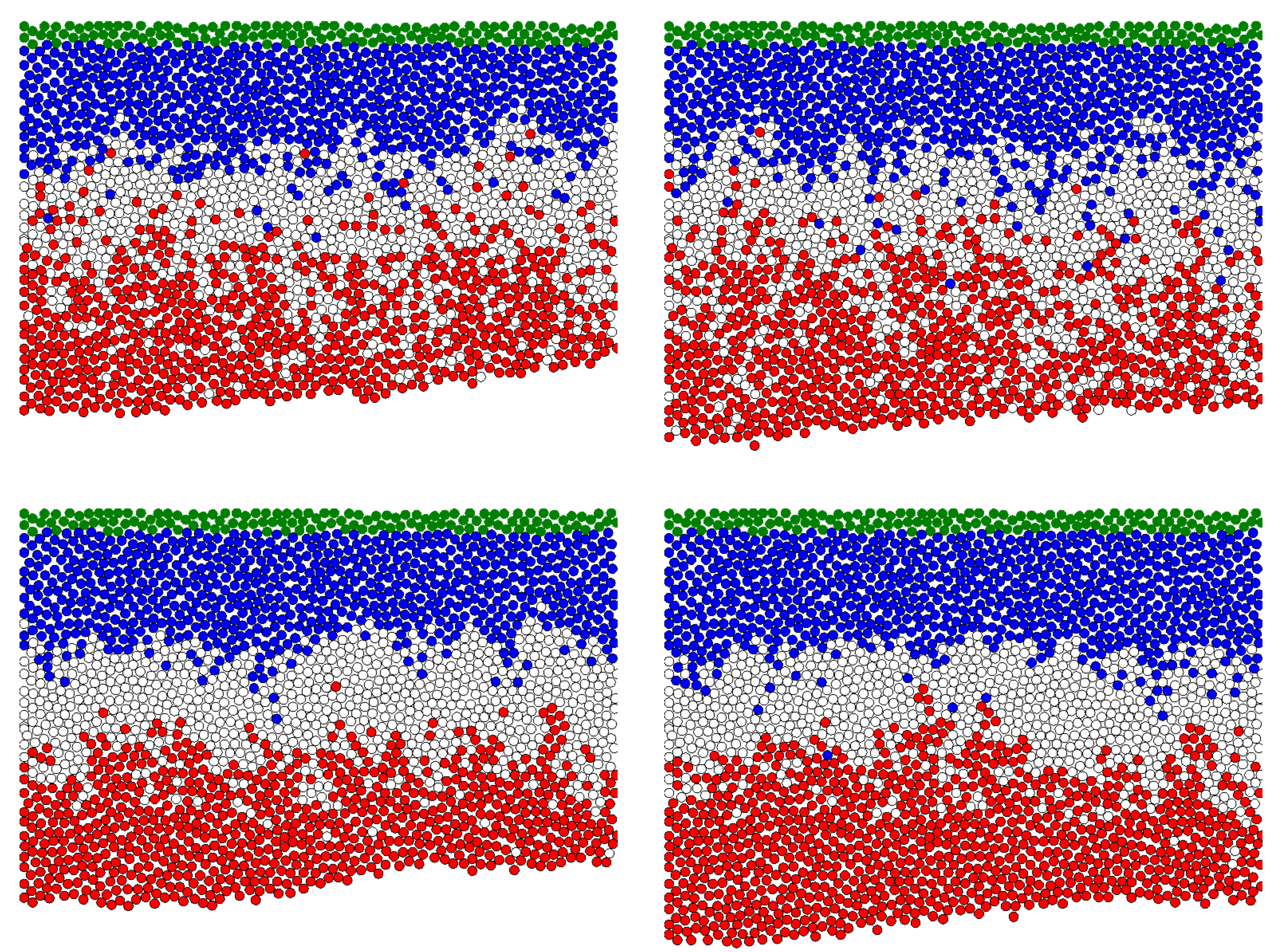

Supplement: S7 Fig — Top weak sorting (left without and right with apoptosis). Bottom medium sorting (left without and right with apoptosis). Apoptosis does not strongly impact the patterning for diffusion-based transport in our simulations. (TIF) [file pcbi.1007417.s008.tif]

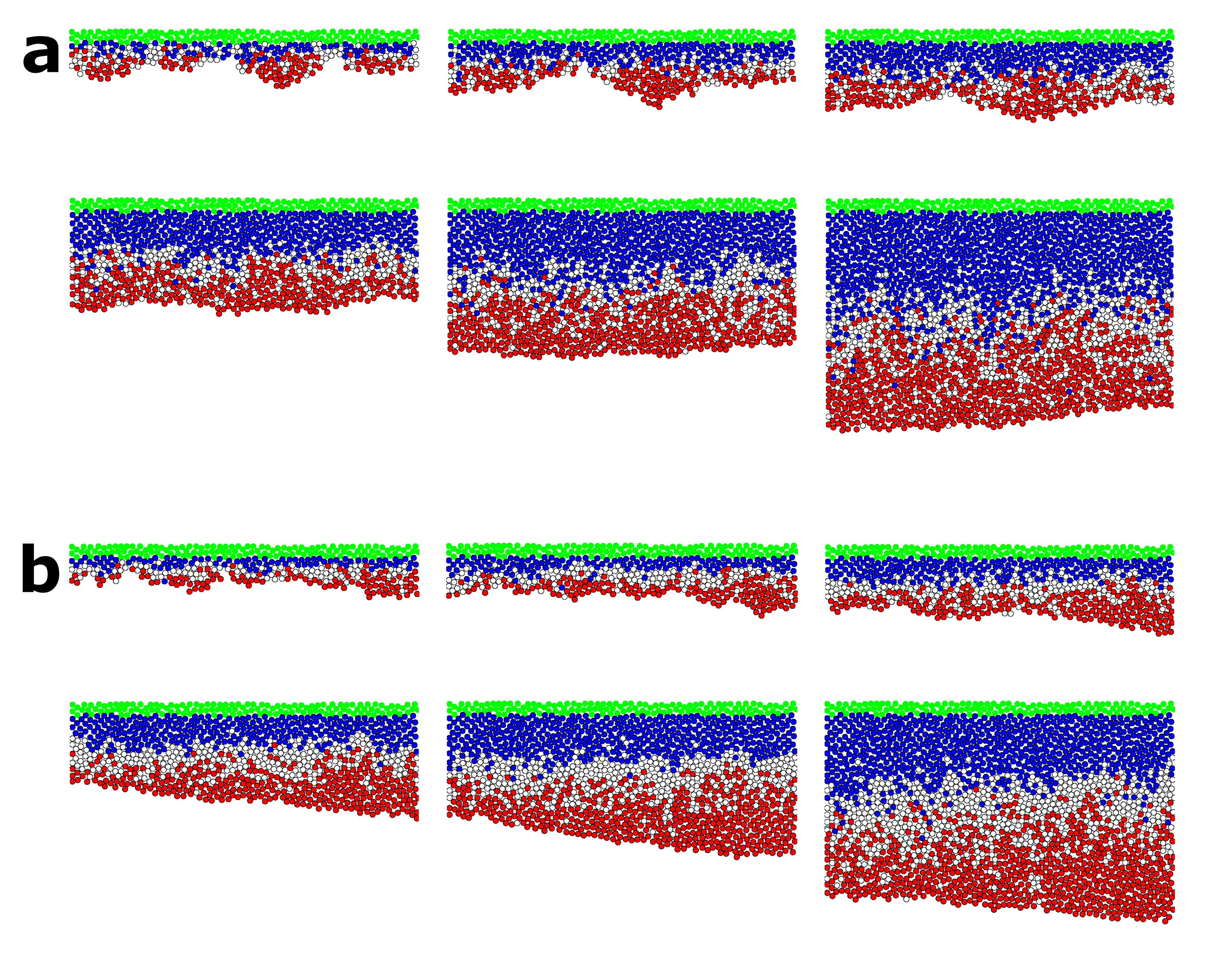

Supplement: S8 Fig — Simulation snapshots of the emerging tissue and its pattern, depicting one exemplary simulation each from Figs 5 and 6. In the top six images diffusion-based transport is shown and in the bottom six images cytoneme based transport is shown. The earlier and more robust establishment of a stable three stripe pattern can be observed in the cytoneme based transport. The thresholds are set to split the tissue into thirds by number at tTRS = 90 min. (TIF) [file pcbi.1007417.s009.tif]

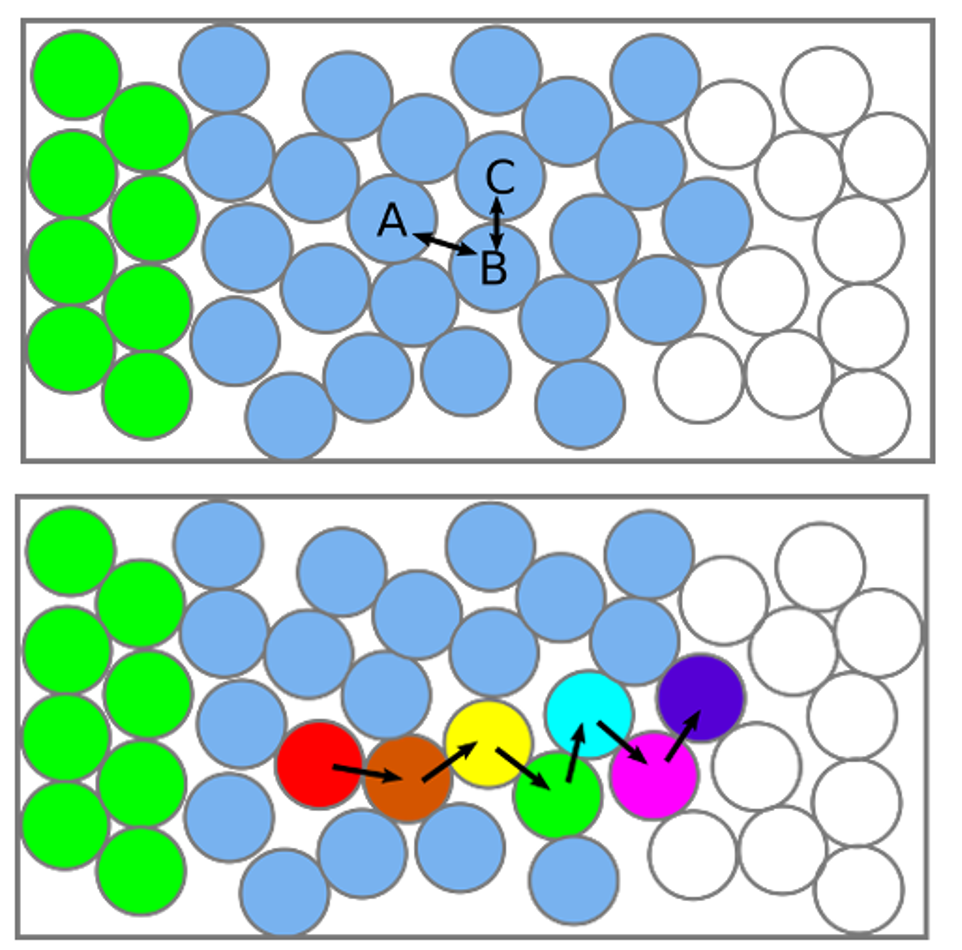

Supplement: S9 Fig — (TIF) [file pcbi.1007417.s010.tif]
